# Chemistry-Informed Machine Learning Enables Discovery of DNA-Stabilized Silver Nanoclusters with Near-Infrared Fluorescence

**DOI:** 10.1021/acsnano.2c05390

**Published:** 2022-09-20

**Authors:** Peter Mastracco, Anna Gonzàlez-Rosell, Joshua Evans, Petko Bogdanov, Stacy M. Copp

**Affiliations:** † Department of Materials Science and Engineering, University of California, Irvine, California 92697, United States; ‡ Chaffey Community College, Rancho Cucamonga, California 91737, United States; § Department of Computer Science, University at Albany-SUNY, Albany, New York 12222, United States; ∥ Department of Physics and Astronomy, University of California, Irvine, California 92697, United States; ⊥ Department of Chemical and Biomolecular Engineering, University of California, Irvine, California 92697, United States

**Keywords:** machine learning, metal nanoclusters, nanomaterials, high-throughput experiments, luminescence

## Abstract

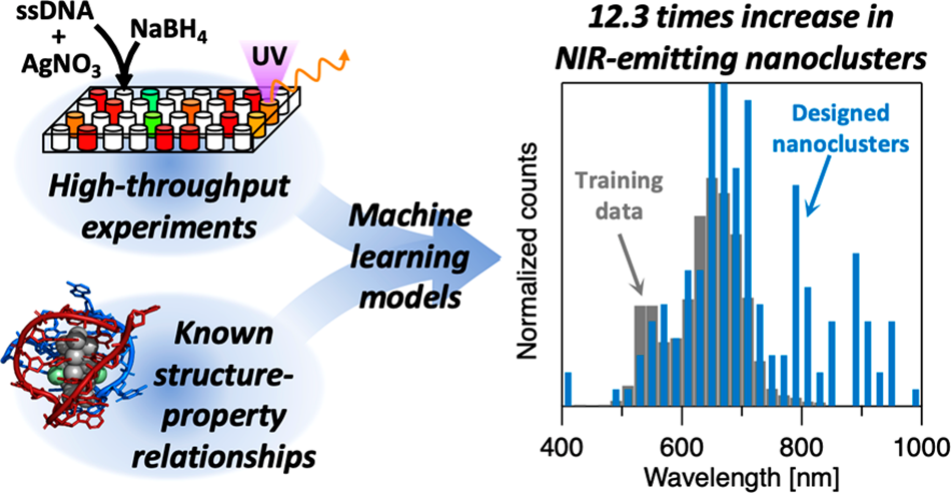

DNA can stabilize silver nanoclusters (Ag_*N*_-DNAs) whose atomic sizes and diverse fluorescence colors are selected by nucleobase sequence. These programmable nanoclusters hold promise for sensing, bioimaging, and nanophononics. However, DNA’s vast sequence space challenges the design and discovery of Ag*_N_*-DNAs with tailored properties. In particular, Ag_*N*_-DNAs with bright near-infrared luminescence above 800 nm remain rare, placing limits on their applications for bioimaging in the tissue transparency windows. Here, we present a design method for near-infrared emissive Ag_*N*_-DNAs. By combining high-throughput experimentation and machine learning with fundamental information from Ag_*N*_-DNA crystal structures, we distill the salient DNA sequence features that determine Ag_*N*_-DNA color, for the entire known spectral range of these nanoclusters. A succinct set of nucleobase staple features are predictive of Ag_*N*_-DNA color. By representing DNA sequences in terms of these motifs, our machine learning models increase the design success for near-infrared emissive Ag*_N_*-DNAs by 12.3 times as compared to training data, nearly doubling the number of known Ag_*N*_-DNAs with bright near-infrared luminescence above 800 nm. These results demonstrate how incorporating known structure–property relationships into machine learning models can enhance materials study and design, even for sparse and imbalanced training data.

## Introduction

Metal nanoclusters represent the smallest of nanoparticles, containing just a few to several hundred metal atoms.^1^ Nanoclusters can be synthesized to atomic precision and possess intriguing photonic properties, such as discrete molecular-like optical spectra and bright luminescence, and these properties depend strongly on nanocluster composition and structure.^2^ To gain control over nanocluster photonics, it is necessary to develop synthetic strategies to control nanocluster structures. A key step in this process is the selection of molecular or atomic ligands, which protect the nanocluster from degradation. Ligands are the architects of metal nanoclusters, controlling the size, geometry, and electronic structure of these atomically precise nanoparticles.^3^ Most frequently stabilized by small molecules like thiolates or phosphines,^4^ noble-metal nanoclusters can also be stabilized by complex macromolecular ligands.^5^ Among these, DNA is an unusually programmable multidentate ligand for noble-metal nanoclusters.^6,7^ Single-stranded DNA can stabilize silver nanoclusters (Ag*_N_*-DNAs) with diverse sequence-selected sizes and visible to near-infrared (NIR) fluorescence colors,^8^ creating a palette of tunable fluorophores that are inherently embedded in DNA. The nanocluster-templating DNA ligands also enable higher-order organization of Ag*_N_*-DNAs^9^ and control near-field nanocluster interactions.^10,11^ Sequence-encoded Ag_*N*_-DNAs present the possibility of achieving atomically precise nanoclusters with programmable structure–property relationships and an inherent biological interface, with potential applications in biosensing, imaging, and integration into versatile DNA nanotechnologies.

Fluorescent Ag_*N*_-DNAs are partially oxidized clusters of *N* = 10–30 silver atoms stabilized by 1–2 DNA oligomers.^12,13^ Ag_*N*_-DNAs possess diverse visible to NIR fluorescence colors. DNA ligands sculpt silver nanoclusters with rodlike shapes,^12,14^ which is a degree of structural anisotropy that is unusual for nanoclusters. This prolate geometry produces a strong correlation of *N* to Ag*_N_*-DNA color^12^ and signatures of plasmon-like excitations,^11,15^ as computationally predicted for nanocluster rods.^16−18^ A dimly emissive violet Ag*_N_*-DNA with a compact shape has also been reported, suggesting that DNA can stabilize either compact or rodlike Ag*_N_*.^13^ Ag_*N*_-DNAs hold significant promise for biosensing,^19^ bioimaging,^20,21^ and molecular logic.^22^ In particular, emerging NIR-emissive Ag_*N*_-DNAs^23−26^ are promising fluorophores for bioimaging in the tissue transparency windows, where biological tissues and fluids scatter, absorb, and emit far less light and suitable fluorophores have been lacking.^27^

However, the science and applications of Ag*_N_*-DNAs have been hindered by the poor understanding of how DNA’s immense sequence space correlates to the diversity of Ag*_N_*-DNA properties. Most researchers stabilize Ag*_N_*-DNAs with oligomers of *L* = 10–30 nucleobases, which have 4^*L*^ possible nucleic acid sequences. While Ag^+^ has a greater affinity for cytosine (C) and guanine (G) than for adenine (A) and thymine (T),^28^ all four nucleobases influence Ag*_N_*-DNA properties.^29,30^ Thus, it is crucial to determine how the sequence encodes Ag_N_ properties and to harness this information to design DNA template sequences for Ag*_N_*-DNAs and other DNA-based nanoclusters.^7,31^

DNA’s combinatorial nature makes machine learning (ML) approaches^32^ well-suited for probing Ag*_N_*-DNA “sequence–structure–property” relationships. Because first-principles models for Ag*_N_*-DNAs are nascent,^33^ experimental data are necessary to enable ML.^30,34−36^ We previously developed high-throughput chemical synthesis and optical characterization^30^ to generate data libraries that connect DNA sequences to visible and NIR fluorescence colors of Ag*_N_*-DNAs.^24,35^ Because Ag*_N_*-DNAs naturally fall into color classes based on magic number properties,^30^ we employed supervised ML to determine how sequence encodes Ag*_N_*-DNA color class. (Supervised ML involves the use of labeled data sets of inputs, e.g. DNA sequence, and their corresponding outputs, e.g. Ag*_N_*-DNA color, to train ML algorithms to map inputs onto outputs. Inputs are represented numerically in the form of feature vectors (features are sometimes called descriptors). The process of choosing which features to use is called feature engineering and is a critical step in ML. Excellent reviews by Ferguson and Domingos provide accessible introductions to ML for readers.^37,38^) Our models were up to 3 times more likely to select 10-base DNA strands for target Ag*_N_*-DNA colors in the visible spectrum as compared to random selection,^35^ and the models remained predictive for DNA strands of other lengths.^39^ However, we were previously constrained to Ag*_N_*-DNAs with fluorescence emission from 450 to 800 nm, limiting the model’s utility for NIR Ag*_N_*-DNAs in the tissue transparency windows. Also, because this work preceded any reports of Ag*_N_*-DNA crystal structures,^14,40^ our models were largely agnostic to Ag*_N_*-DNA structure–property relationships and required naïve data mining for feature engineering, resulting in models with high dimensionality and limited interpretability.^35,39^

Emerging Ag*_N_*-DNA crystal structures provide critical insights into how DNA oligomers stabilize Ag_*N*_. Others have reported the structures of a green-emissive nanocluster stabilized by 6-base oligomers^40^ and of several NIR-emissive Ag_16_-DNAs stabilized by variations of a 10-base oligomer.^14,41,42^ We hypothesize that information from these crystal structures can improve ML prediction of Ag*_N_*-DNA color and enable the discovery of NIR Ag*_N_*-DNAs, even though there are far fewer available training examples for NIR Ag*_N_*-DNAs^24^ as compared to visibly fluorescent Ag*_N_*-DNAs.^35,39^ To test this hypothesis, we construct feature vectors enumerating nucleobase “staple” features that capture aspects of DNA–silver interactions in the crystal structures. We also dramatically expand our training data’s spectral window by including recently discovered NIR Ag*_N_*-DNAs with peak emission up to 1000 nm^24^ and construct an ML model that is well-suited to limited and imbalanced training data. Our chemically informed approach increases the likelihood of obtaining target Ag*_N_*-DNA colors by up to 10-fold. Furthermore, feature analysis uncovers nucleobase staple features that strongly discriminate between Ag*_N_*-DNA color classes, providing insights into how DNA oligomers coordinate Ag_*N*_. This work shows that incorporating known information about structure–property relationships in the feature engineering process and addressing imbalanced training data through data sampling can significantly improve ML model performance and interpretability and, in turn, improve design success, even for sparse nanomaterials data sets and rare classes.

## Results and Discussion

**Figure 1:**
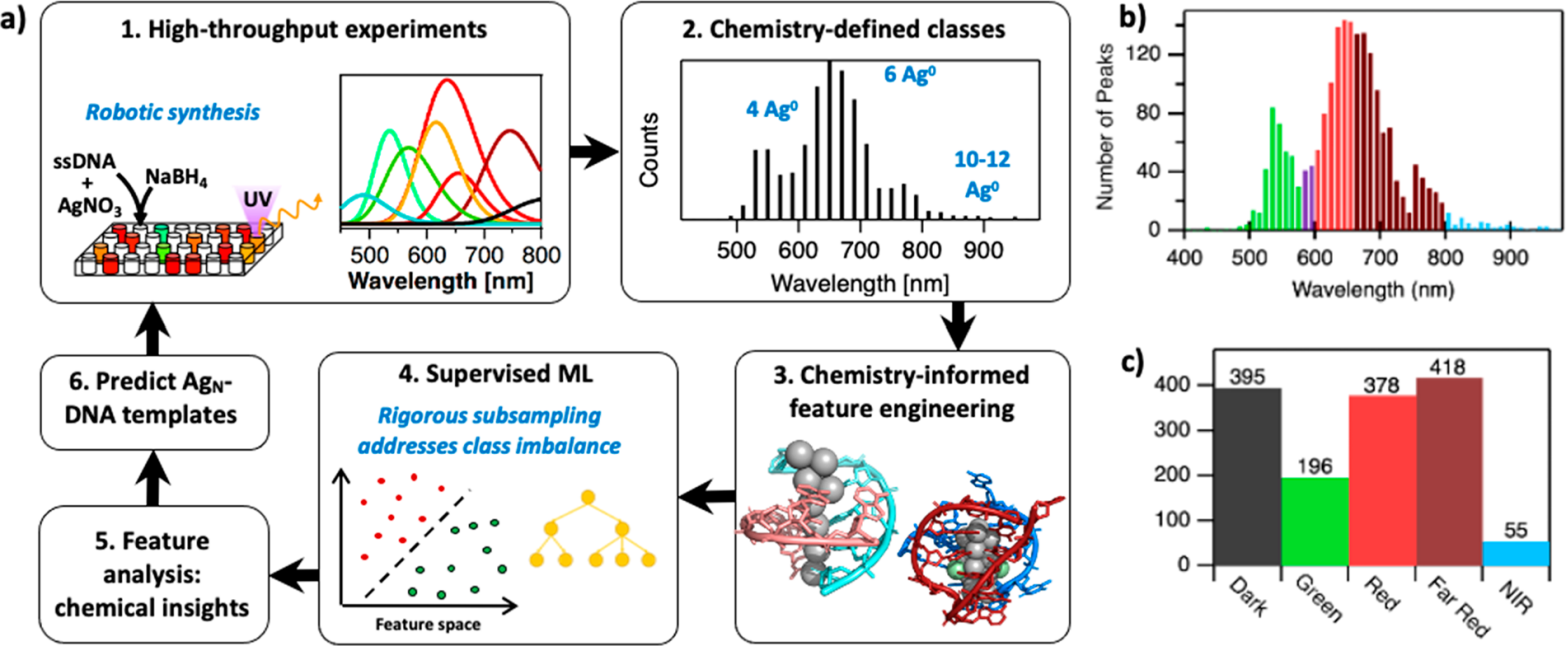
Workflow and training data for Ag_*N*_-DNA color prediction. (a) Schematic of the workflow for ML-enabled Ag*_N_*-DNA discovery (PDB accession codes 6NIZ(40) and 6JR4(14)). (b) Histogram of training data values of Ag*_N_*-DNA peak wavelength, λ_p_. Colors indicate the boundaries of Green (green), Red (red), Far Red (dark red), and NIR (blue) classes. Purple bars represent λ_p_ values of sequences omitted from the training data, as the magic numbers of Ag*_N_*-DNAs in this region are unknown. (c) Class sizes for the five Ag*_N_*-DNA color classes.

The goals of this study are to determine the DNA sequence attributes that select Ag*_N_*-DNA fluorescence colors and to experimentally validate the saliency of this chemical information by designing DNA template sequences for specific Ag*_N_*-DNA colors. We also aim to significantly expand the spectral window of Ag*_N_*-DNA ML models to enable the discovery of NIR-emissive Ag*_N_*-DNAs. Figure 1a illustrates the workflow of this study. First, we assemble a training data library of UV-excited fluorescence emission spectra of Ag*_N_*-DNA products stabilized by 2661 10-base oligomers (representing 0.25% of all possible 10-base sequences), from past high-throughput experiments.^24,35,39^ These spectra have been fitted to a sum of one to three Gaussians as a function of energy to determine the Ag*_N_*-DNA emission peak(s) associated with each DNA sequence, and products are considered “bright” if peak area is above a specific defined threshold, as in past work^24,35,39^ (details in Sections 1.1 and 2.2 in the Supporting Information). We solely use this data library because the high-throughput experiments were performed with consistent stoichiometry and robotic pipetting methods, and the resulting Ag*_N_*-DNA products were reported for *all* sequences, unlike the majority of studies that do not report DNA sequences that were not suitable templates for Ag*_N_*-DNAs.^43^ Moreover, Swasey et al. reported 162 10-base oligomers with peak emission >750 nm, motivating the focus on 10-base oligomers. (Our past study showed that ML classifiers trained on 10-base oligomers were also predictive of Ag*_N_*-DNA color for other oligomer lengths,^39^ and it is possible that similar methods could be used to expand the ML model presented here to Ag*_N_*-DNA templates beyond 10-base oligomers.)

The distribution of peak emission wavelengths, λ_p_, for this data set has multiple modes in the visible range (Figure 1b). These modes arise from Ag*_N_*-DNA structure–property relationships, including the strong correlation of cluster size to λ_p_^12^ and the enhanced stabilities of Ag*_N_*-DNAs with magic numbers of neutral silver atoms, *N*_0_. These produce distinct “magic color” classes of Ag*_N_*-DNAs: green-emissive Ag*_N_*-DNAs containing *N*_0_ = 4 neutral silver atoms per cluster,^30,44^ red-emissive Ag*_N_*-DNAs containing *N*_0_ = 6, and NIR-emissive Ag*_N_*-DNAs containing *N*_0_ = 10–12.^24,30^ The step function at 750 nm (Figure 1b) is an artifact of sourcing data from two instruments. A custom plate reader for NIR fluorescence emission has a higher sensitivity^45^ than the commercial plate reader used at lower wavelengths.^30^ Experiments performed with the NIR plate reader also used a slightly increased AgNO_3_ concentration to enhance the chemical yield of larger, NIR Ag*_N_*-DNAs.^24^ Because Swasey et al. reported Ag*_N_*-DNA wavelengths >750 nm with this method, the inclusion of these NIR training data leads to the step function at 750 nm. Apart from this difference, all training data were collected using identical robotic synthesis methods and normalized to one control Ag*_N_*-DNA, allowing direct comparisons of fluorescence brightness and λ_p_ among all samples^35,39^ (details in Methods).

### Color Class Definitions

We employ supervised ML classification to discriminate DNA sequences associated with distinct Ag*_N_*-DNA “color classes.” A classification approach is motivated by Ag*_N_*-DNA structure–property relationships, with color classes defined based on known magic number sizes^30^ or other apparent modes in the λ_p_ distribution.^35,39^ As described below, DNA sequences are categorized by λ_p_ of the brightest spectral peak: “Green” defined as λ_p_ < 580 nm, “Red” as 600 nm < λ_p_ < 660 nm, “Far Red” as 660 nm < λ_p_ < 800 nm, and “NIR” as λ_p_ > 800 nm (Figure 1b). Sequences correlated with no measured fluorescence are categorized as “Dark”. In our past work, the wavelength cutoff between Green and Red was chosen because these Ag*_N_*-DNAs have distinct magic numbers of *N*_0_ = 4 and *N*_0_ = 6, respectively.^30^ Sequences whose brightest peak is 580 nm < λ_p_ < 600 nm are excluded from training data because *N*_0_ is currently unknown in this range. The cutoff between Red and Far Red was chosen based on the shape of the λ_p_ distribution from 600 to 700 nm, which suggests distinct types of nanocluster structures.^35^

With the expansion of the training data set up to λ_p_ = 1000 nm,^24^ it is necessary to define a NIR color class beyond Far Red. Ag*_N_*-DNAs with *N*_0_ = 6 are reported up to λ_p_ = 685 nm, and *N*_0_ = 10–12 Ag*_N_*-DNAs are reported with λ_p_ = 775–1000 nm.^24,46^ Because *N*_0_ values are unknown for λ_p_ = 685–775 nm, it is not possible to define the cutoff between Far Red and NIR with known structure–property information. Instead, we used statistical methods to select this cutoff. First, we applied *k*-means clustering to the set of all λ_p_ values. (*k*-means clustering is a form of unsupervised ML that learns to group data points into discrete “clusters” that contain data that are more similar to one another than to data in other clusters.^37^) This method yielded four distinct clusters with centroids at 547, 637, 687, and 797 nm; midway points between cluster centroids are at 592, 662, and 742 nm (see Section 2.1 and Figure S1 in the Supporting Information). This supports the existence of four color classes, and the midway points between centroids align well with the previously defined cutoffs for Green/Red and Red/Far Red. Therefore, we retain the previous definitions of “Green” as λ_p_ < 580 nm and “Red” as 600 nm < λ_p_ < 660 nm, with peaks from 580 to 600 nm omitted from training data due to a lack of information about the magic number *N*_0_ in that regions.^35^ However, the step function artifact in Figure 1b is likely to obscure the natural Ag*_N_*-DNA color distribution for λ_p_ > 750 nm. For this reason, we then tested Far Red/NIR cutoffs from 720 to 800 nm, comparing 10-fold cross-validation accuracies of support vector machines (SVMs) trained to distinguish Far Red and NIR sequences, as described below. The accuracy was highest for a cutoff of 800 nm (Figure S2). Because cutoffs above 800 nm dramatically diminish the NIR class and caused overfitting, we assign λ_p_ = 800 nm as the Far Red/NIR cutoff.

To best determine how DNA sequence encodes Ag*_N_*-DNA color, we exclude from training data all sequences producing multiple bright peaks in two or more color classes. These sequences represent DNA strands that can adopt multiple different conformations around Ag*_N_*-DNAs of different compositions and are likely to combine patterns associated with multiple Ag*_N_*-DNA colors. (Such “multi-colored” sequences may be of relevance for Ag*_N_*-DNAs used in color-switching sensing schemes.^19^) This combination of nucleobase patterns associated with multiple Ag*_N_*-DNA colors may complicate feature engineering and ML, which is why these sequences are excluded from training. Sequences with mediocre fluorescence brightness are also excluded (details in Methods and Section 2.3 in the Supporting Information). With these definitions, we distill a training data set of 1443 sequences sorted into Green, Red, Far Red, NIR, and Dark classes. Notably, class sizes are highly imbalanced for this data set (Figure 1c), a factor that we address below.

### ML Classifier Ensemble

We choose SVM classifiers for this study. For *n*-dimensional samples from two classes, this supervised ML method learns an (*n* – 1)-dimensional hyperplane that separates the two classes. The class of an unseen data point is predicted based on its location relative to the fitted hyperplane.^37^ As before,^34,35^ here we found that SVMs perform comparably to or better than similar and more complex ML algorithms in discriminating Ag*_N_*-DNA color classes and have a lower computational training cost. For this study, we choose SVMs with L1 regularization that naturally performs feature selection.^47^

**Figure 2:**
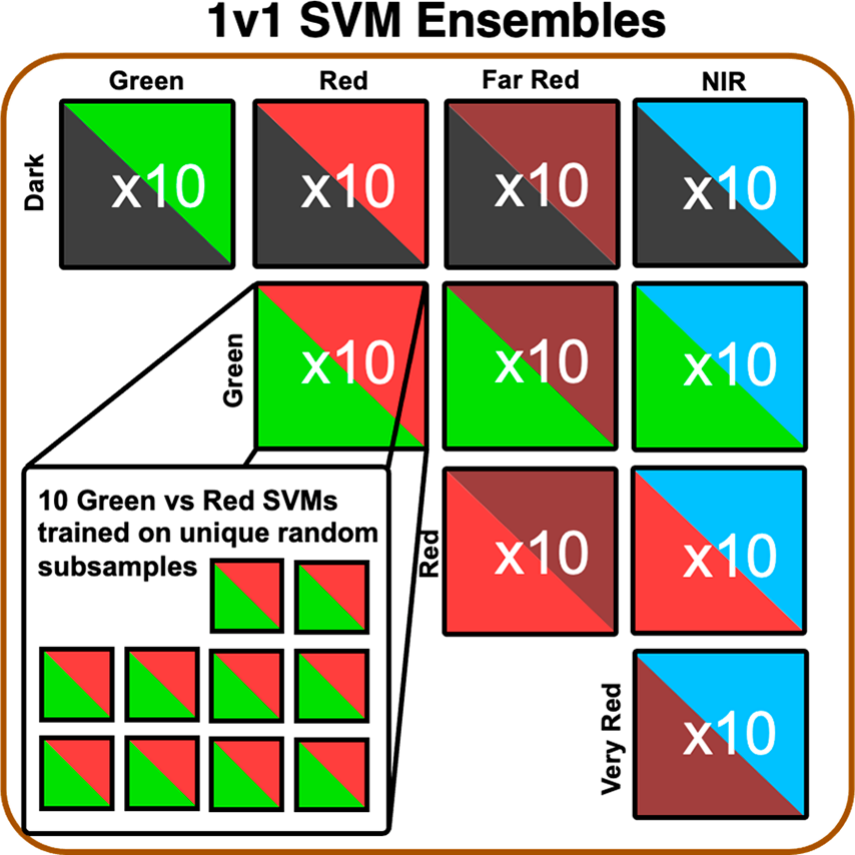
ML classifier ensemble architecture. Schematic of the ML classifier ensemble model used to discriminate DNA sequences in Dark, Green, Red, Far Red, and NIR classes. The ensemble consists of 10 sets of 10 SVMs, with each set corresponding to a pair of color classes. Each SVM is trained on a different random balanced subset of the training data for the given pair of color classes. For an input sequence, the consensus of all trained SVMs is used to determine the most likely color class.

ML classifiers trained on imbalanced data sets will favor the dominant class, severely limiting predictive power for the minority class.^48^ Because nanomaterial data sets are often naturally imbalanced, ML models for nanomaterial prediction should rigorously address class imbalance.^48^ In this case, we have nearly 10 times fewer NIR sequences than Far Red sequences (Figure 1c), which significantly challenges the discovery of NIR Ag*_N_*-DNAs. For this reason, we construct an ensemble ML classification approach that is effective for imbalanced experimental data sets of limited size.^49^ Our model consists of 100 individual “one-versus-one” (1v1) classifiers trained to discriminate between possible pairs of Green, Red, Far Red, NIR, and Dark classes (Figure 2) (1v1 classifiers generally perform better than multiclass classifiers for small data sets). For each pair of color classes, 10 distinct classifiers are trained on data sets balanced by different random subsamples of the larger class. The average consensus of these 100 classifiers is then used to predict the color class of unseen sequences, addressing class imbalance without sacrificing sensitivity to data trends.

### Feature Engineering

**Figure 3:**
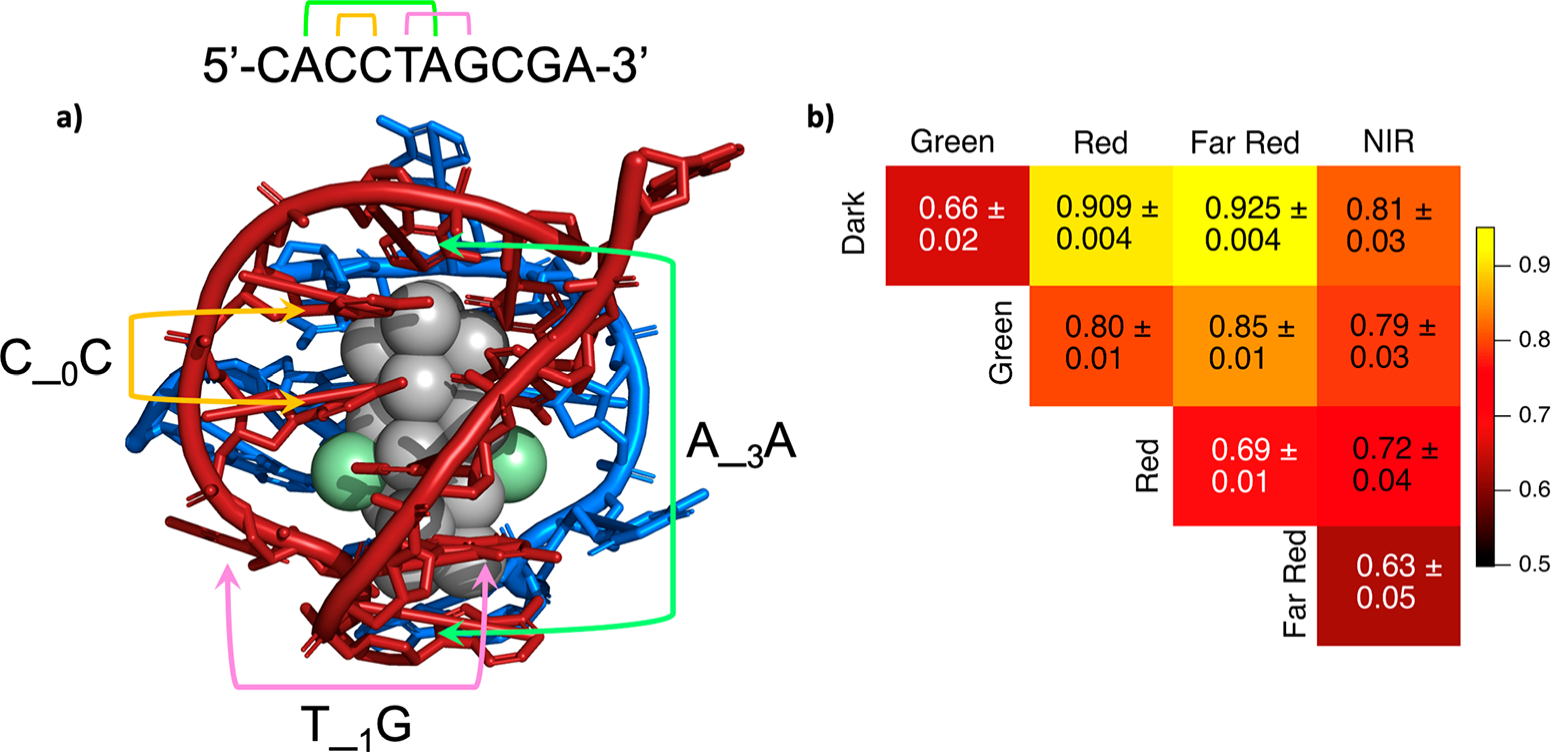
(a) Staple nucleobase motifs, illustrated for a crystal structure of an Ag_16_-DNA reported by Cerretani et al. (PDB accession code: 6JR4),^14^ composed of two 10-base DNA oligomers (red and blue), 16 silver atoms with occupancy 1 (gray), and 2 silver atoms with low occupancy 0.31–0.36 (green). Brackets illustrate nucleobase staple features that capture critical aspects of DNA–silver interactions involved in Ag*_N_*-DNA stabilization, including adjacent Cs that stabilize the long sides of the Ag_16_ (yellow), nonadjacent As that cap ends of the Ag_16_ (green), and nonadjacent T and G that appears important for promoting DNA flexibility as the strand curves around the end of the Ag_16_ (pink). (b) Average 10-fold cross-validation scores for each 1v1 classifier SVM ensemble trained using feature vectors of nucleobase staple features.

ML requires a choice of input data representation, or “feature vectors”. Learning is most effective when features capture properties of the trend one seeks to learn.^50^ For many nanomaterial systems, this information is unknown.^32^ Previously, we used naive data mining^35^ to engineer ∼200-component feature vectors that indicated occurrences of select color-correlated sequence motifs of up to seven adjacent nucleobases. These feature vectors had several drawbacks, including redundancy of many motifs. To simultaneously improve ML efficacy and use the ML process to advance the fundamental understanding of Ag*_N_*-DNAs, here we design feature vectors based on chemically motivated observations. Consider the crystal structure of the rod-shaped Ag_16_ stabilized by two copies of a 10-base oligomer (Figure 3a).^14^ In this Ag_16_-DNA, pairs of both adjacent *and* nonadjacent nucleobases facilitate key nanocluster–DNA interactions. For example, the Ag_16_ rod’s long sides are protected solely by adjacent Cs and Gs (e.g. orange bracket, Figure 3a), suggesting that CC, CG, GC, and/or GG are important for protecting lower curvature faces of Ag_*N*_. In contrast, a pair of nonadjacent As at positions 2 and 6 of one strand protect Ag_16_ ends (green bracket, Figure 3a), together with the second strand’s C at position 1 and A at position 6. The T at position 5 illustrates the importance of nucleobases that promote DNA strand flexibility; this nucleobase is unbound to the Ag_N_ but enables the DNA to bend around the end of the Ag_N_ (pink bracket, Figure 3a). Based on this structure, we hypothesize that feature vectors representing both adjacent *and* nonadjacent nucleobase patterns are important for the stabilization of Ag*_N_*-DNAs.

We choose the simple representation X__*m*_Y to quantify the prevalence of all pairs of nucleobases X and Y separated by *m* arbitrary nucleobases, *m* = 0, 1, ..., 8. We refer to X__*m*_Y as nucleobase “staple” features, representing two distinct nucleobase ligands that coordinate the Ag_*N*_ at zero, one, or two sites. The term “staple motif” is used to describe ligand–metal units that are commonly found at the surface of monolayer-protected nanoclusters, in which two or more surface metal atoms are bridged by two ligands.^51,52^ In analogy, certain pairs of nucleobases X__*m*_Y protect the Ag_*N*_ at two sites. For example, C__0_C represents the motif stabilizing the upper left side of the Ag_16_, while A__3_A represents the motif that stabilizes cluster ends (Figure 3a). We test feature vectors whose components count occurrences of all 144 possible X__*m*_Y features in a sequence (note that we do not only cherry-pick base patterns from the single-crystal structure in Figure 3a). Because staple features are positionally independent, i.e. encode no information about the position of X__*m*_Y in a sequence (except for X__8_Y, which represents 5′- and 3′-ends), we also consider feature vectors of location-specific nucleobase information by “one-hot encoding,” representing a 10-base sequence as a length-40 vector (Figure S3).

### Feature Analysis

To gain insights into how the DNA sequence encodes the Ag*_N_*-DNA color, we use feature analysis, whereby features are selected or ranked by their impacts on ML model performance.^53^ Because ML classifiers are more accurate when features encode information that is relevant to the trend the classifier is tasked to learn, variations in a model’s accuracy for different choices of features can be used to discern which features are most important for classification. We first compare 10-fold cross-validation accuracies of the model in Figure 2 trained using three different feature vectors: (1) only nucleobase staple features, (2) only one-hot encoding features, and (3) the combination of both features. One-hot encoding represents the exact positions of each nucleobase within the strand (example in Figure S3), while nucleobase staple features represent the relative positions of pairs of nucleobases (example in Figure 3a). The model’s accuracies for only one-hot encoding (Figure S5) are lower than for only nucleobase staple features (Figure 3b), especially for pairwise SVMs that included the NIR class (all scores shown in Figures S5–S7). Thus, this result supports the hypothesis that the relative positions of nucleobases with respect to one another are more important than exact nucleobase locations in a strand for determining if and how a 10-base strand stabilizes Ag_*N*_. Because feature vectors combining staple features and one-hot encoding (Figure S7) do not increase accuracies compared to staple features alone, we use the lower-dimensional nucleobase staple features only for the studies below.

We next investigate how staple features select Ag*_N_*-DNA color, using feature selection to score features based on their importance for random forest classifier accuracy relative to randomly generated “shadow features,” or meaningless inputs (details in Methods). (Random forest is an ensemble learning method consisting of many distinct decision trees, where the collective predictions of the decision trees are used to determine the model’s output.) This approach has provided insights into nanomaterial synthesis conditions^54^ and methane uptake by metal–organic frameworks.^53^ For each pair of color classes, at most 16 of the 144 staple features scored higher than the most important shadow feature: i.e., sufficiently higher than random. The union of all staple features that scored higher than random for the 10 color class pairs is a set of 23 staple features. To verify that these are predictive of Ag*_N_*-DNA color, we trained 1v1 SVMs using feature vectors of the top *n* staple features ranked by importance score. For all color class pairs, SVM accuracies plateau for feature vectors of only “important” staple features (Figure S9), supporting the particular relevance of these 23 motifs for Ag*_N_*-DNA color selection.

**Figure 4:**
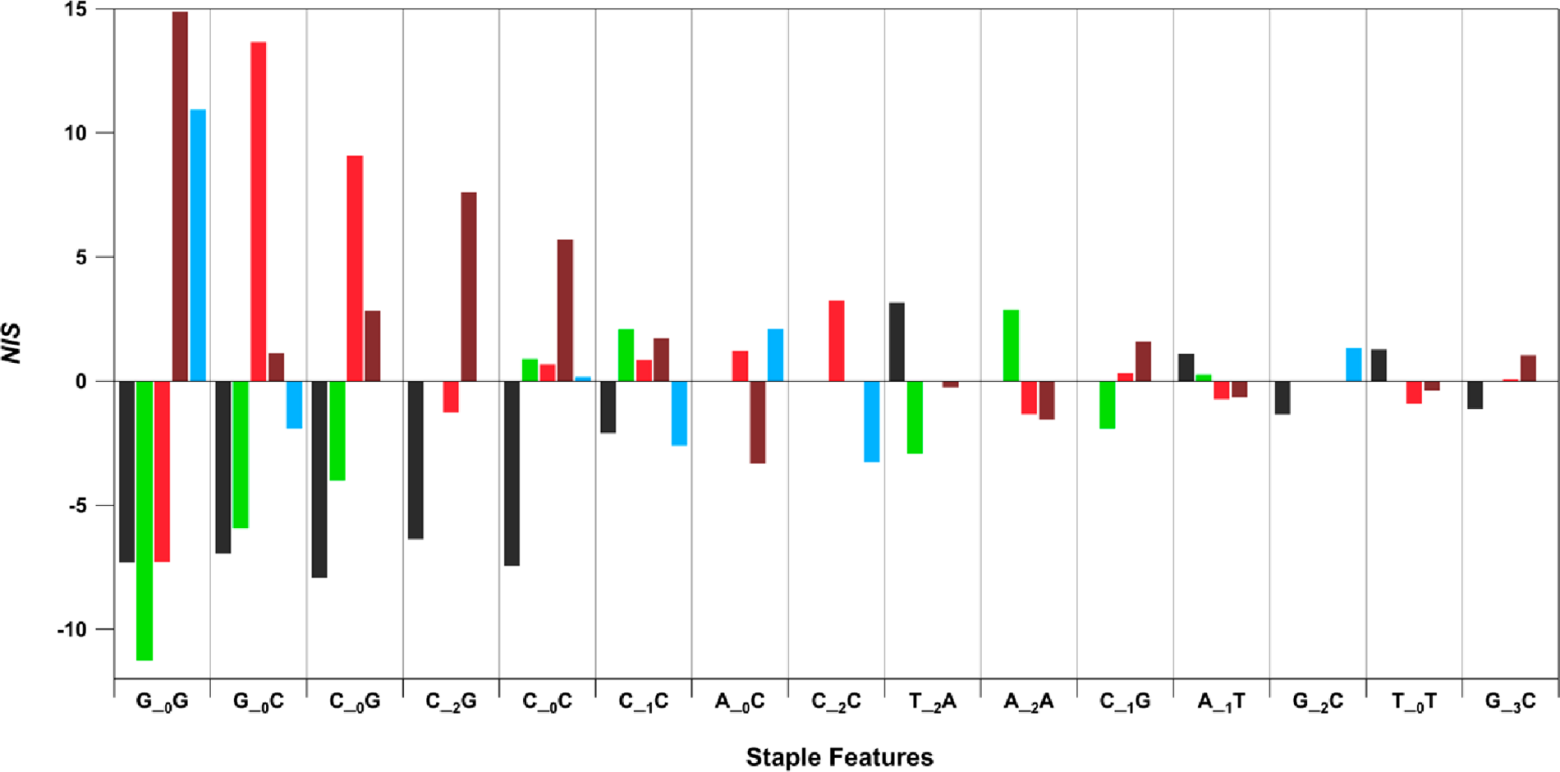
Color-correlated staple features. Net importance scores (*NIS*) of the top 15 ranked staple features. Each colored bar corresponds to a distinct color class: Dark (black), Green (green), Red (red), Far Red (dark red), and NIR (blue).

The feature selection method we implement assigns scores in the context of 1v1 classifiers. To determine a staple feature’s importance for a single color class, we define a net importance score (*NIS*) that combines all four importance scores for a specific motif and a specific color class (defined in Note 1 in the Supporting Information). *NIS* > 0 represents an overall positive correlation between a motif and a color class, and *NIS* < 0 represents an overall negative correlation. Figure 4 displays *NIS* for the 15 staple features with the highest values of |*NIS*| (all scores in Figure S10). These motifs heavily feature C and G, agreeing with past findings that sufficient C and G content is needed to stabilize fluorescent Ag*_N_*-DNAs.^8^ As we found before,^35^ consecutive *G*′s are the single strongest determinant of larger, longer wavelength Ag_*N*_. G__0_G strongly favors Far Red and NIR and disfavors Dark, Green, and Red. G__0_C and C__0_G are less selective for high wavelength Ag*_N_*-DNAs, favoring Red and disfavoring Dark and Green. Figure 4 also illustrates the collective importance of multiple staple features in selecting Ag*_N_*-DNA color. For example, C__0_C only selects against Dark, with *NIS* > 0 for all fluorescent color classes. While Far Red is most strongly correlated with C__0_C, other staple features are needed to determine the exact Ag*_N_*-DNA atomic size/structure. Figure S11 compares the relative abundance of all 144 staple features in the five color classes, showing a rich and complex dependence on many of the staple features. The complexity of Ag*_N_*-DNA sequence–structure–property relationships points to the utility of ML models for Ag*_N_*-DNA design. ML models better capture collective effects of multiple staple features on Ag*_N_*-DNA stabilization than a small set of empirical rules. Future crystallographic studies of Ag*_N_*-DNAs may shed further light on the roles of the motifs in Figure 4.

### Ag*_N_*-DNA Ligand Sequence Design

To experimentally validate the saliency of staple features for determining Ag*_N_*-DNA color, we use the ML model to design the sequences of 10-base DNA ligands for stabilizing Green, Far Red, and NIR Ag*_N_*-DNAs. These classes were chosen for testing because their design is likely to be the most challenging. This greater challenge is expected because (i) Green and NIR are the least abundant color classes in the training data and (ii) class imbalance is greatest between Far Red and NIR (Figure 1c). Our SVM-based model’s low computational cost allows us to rapidly train the model and then predict Ag*_N_*-DNA color for all 4^10^ 10-base DNA sequences. The model was trained using all available training data (i.e., no data reserved for cross-validation) in 7.8 s on an AMD Ryzen 9 5950X 3.4 GHz Core-Processor, followed by assigning average SVM scores to all 4^10^ sequences in 12 min. (This is a significant increase in speed as compared to our prior models, for which it was infeasible to assign predictions for all 4^10^ 10-base DNA sequences.^34,35,39^) For each target color class, sequences are scored by the minimum average probability of falling into the target class for the four relevant color class pairs. For example, a sequence’s likelihood of being Green is assigned as the minimum average Green probability from the SVMs for Green vs Dark, Green vs Red, Green vs Far Red, and Green vs NIR (average probability computed from the 10 SVMs associated with each pair of color classes). This scoring preferentially ranks sequences by likelihoods of *not* falling into any undesired class. The top 124 sequences for each target color are then experimentally tested by methods identical to training data collection (see Methods and Section 1 in the Supporting Information).

**Figure 5:**
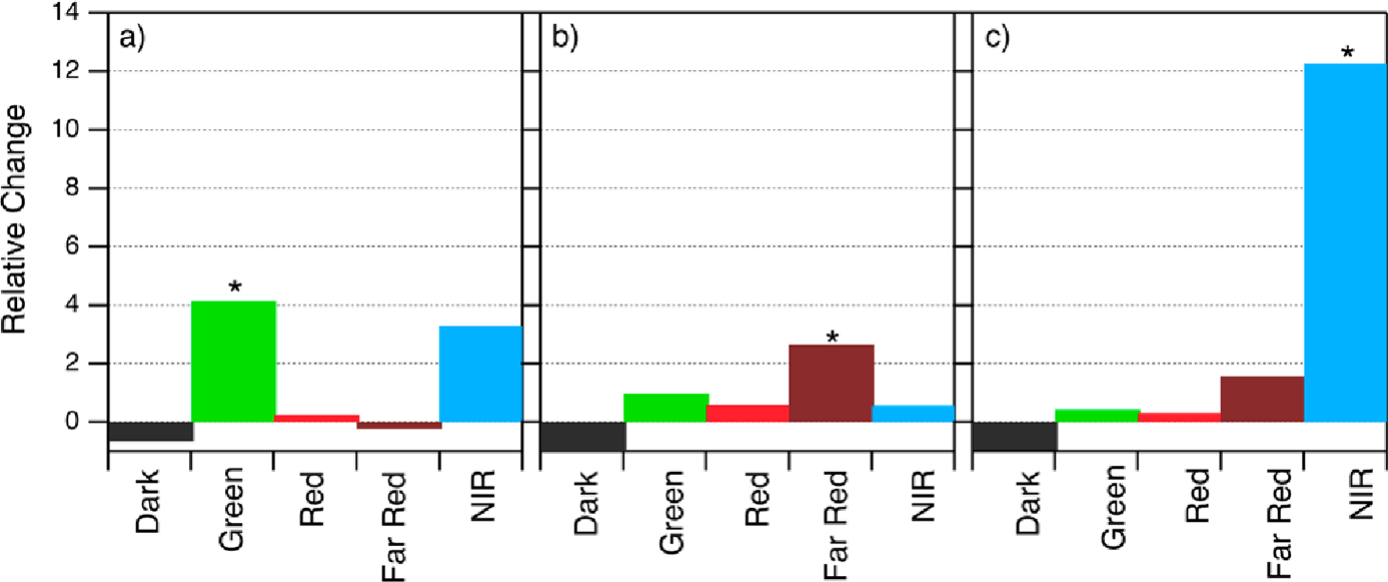
Relative change of each class size for (a) Green-designed sequences, (b) Far-Red-designed sequences, and (c) NIR-designed sequences. In each case, ML-aided sequence selection results in the greatest relative increase in the target color class (asterisks) as compared to all other classes. Far-Red-designed sequences (b) result in no Dark sequences, and the high selectivity against NIR sequences for Far-Red-designed sequences is notable because the class imbalance is greatest between Far Red and NIR classes (Figure 1c).

In all three design cases, the target color experiences the greatest relative change of fractional size as compared to training data (Figure 5). This model increases the fraction of Green sequences by a factor of 4 as compared to the training data and significantly outperforms our past model’s relatively low selectivity for Green Ag*_N_*-DNAs by 5.9 times. This result is particularly notable given the previously identified challenge of distinguishing Green from Dark.^35^ We also find that 11 of the Green-designed strands produced NIR Ag*_N_*-DNAs, including the longest-wavelength Ag*_N_*-DNA reported to date, with λ_p_ = 1041 nm. Five of these 11 Green-designed strands produce both NIR products and products with emission ≤ 583 nm. Further studies may illuminate whether Green Ag*_N_*-DNA template sequences share features of NIR Ag*_N_*-DNA template sequences.

Far Red design produces the greatest fraction of sequences in the target color class, with 60% experimentally determined to be Far Red (Figure S13). The relative increase in Far Red sequences is less than for Green (Figure 5a,b), which is expected because Far Red is the largest class in our training data (Figure 1c). Notably, Far Red design is also highly selective against several other color classes. No designed Far Red sequences are Dark, and only five are NIR, despite the greatest class imbalance between Far Red and NIR.

Selectivity for NIR is especially high. While NIR sequences represent only 2% of the initial training data (55 of 2661 sequences), their prevalence increases to 27% by ML-guided design (Figure 5c), for a total of 34 NIR Ag*_N_*-DNAs discovered among the NIR-designed sequences. Combined with the 16 NIR Ag*_N_*-DNAs identified among Green- and Far-Red-designed sequences (color distributions in Figure S14), our findings nearly double the number of known Ag*_N_*-DNAs with λ_p_ > 800 nm,^24,29^ expanding the number of these fluorophores by 90%. This significant expansion of Ag*_N_*-DNAs in the tissue transparency windows provides additional candidates for NIR fluorophores for bioimaging. It is particularly important to have a sufficient number of Ag*_N_*-DNA species with NIR spectral properties in order to develop these emitters into NIR biolabels, as their additional important properties, including chemical and photostability, can vary by Ag*_N_*-DNA species and are also not well-studied. Development of NIR Ag*_N_*-DNA biolabels for fluorescence imaging is ongoing and is outside the scope of this work. Our results also experimentally support the relevance of the identified staple features for selecting Ag*_N_*-DNA color, as well as the effectiveness of statistical sampling and classifier ensembles for limited data sets with rare classes. The ML model presented here may also be adapted to predict other properties of nucleic acid based nanoclusters, such as sensitivity to analytes^19^ or catalytic behavior, as was recently reported for Ag*_N_*-DNAs.^55,56^

## Conclusions

We have presented a ML model that combines limited experimental data with recent crystallographic insights to capture the sequence–structure–property relationships of Ag*_N_*-DNAs. This model employs significantly lower dimensional features than previous ML models for Ag*_N_*-DNAs and accounts for training data imbalance through statistical sampling and classifier consensus. We also use the model to provide insights into how DNA strands select Ag*_N_*-DNA sizes and colors. Certain nucleobase staple features play significant roles in determining Ag*_N_*-DNA fluorescence color, and these motifs may inform an understanding of the DNA–silver interaction in Ag*_N_*-DNAs. Furthermore, the model’s predictive power is experimentally verified, increasing the prevalence of target Ag*_N_*-DNA color classes by up to 12.3 times. Our findings provide a design tool for DNA template sequences for Ag*_N_*-DNAs, with special utility for the discovery of NIR Ag*_N_*-DNAs with fluorescence in the tissue transparency windows for applications in bioimaging. The ML methods developed here have broad applicability for sequence-encoded biomolecules, where experimental training data may be limited and challenging to obtain.

## Methods

### Training Data Curation

Training data were sourced from our past high-throughput experiments. These experiments used identical synthesis procedures and the same fluorescence excitation light source. Data are freely available in open-access Supporting Information of past publications and compiled in Supporting Data Files in the Supporting Information, according to best practices for ML in chemical sciences.^57^ All 2661 DNA sequences were correlated to their associated Ag*_N_*-DNA emission spectra collected in the visible spectral region and up to 800 nm.^24,35,39^ NIR fluorescence emission information was compiled from Ag*_N_*-DNAs discovered by Swasey et al.,^24^ using a custom well plate reader with a 675–1325 nm spectral range.^45^ This data set is available as Supporting Data 1 in the Supporting Information and includes fit values for all peaks, including those above and below the defined brightness threshold. Finally, sequences were sorted into the color classes defined in the main text, and this distilled data set of 1443 sequences was used to train ML classifiers.

### Machine Learning Classifier Ensemble

Support vector machines (SVMs) were implemented using the Python scikit-learn package.^58^ The linearSVC module with L1 regularization was used due to the limited size of the training data set, and a regularization parameter of *c* = 0.1 was chosen (Figure S4). For each 1v1 classifier, the more abundant color class was randomly subsampled to balance class size. Classifier performance was assessed by 10-fold cross-validation, which splits training data into 10 folds, using 9 folds for training and 1 fold to assess classifier accuracy, and averages the accuracy from these 10 trained classifiers. For each 1v1 classifier, we performed this process 100 times, averaging over 100 different random choices of the 10 folds, to capture the natural variability that occurs due to subsampling for class balancing. Details are provided in Section 2.6 in the Supporting Information.

### Feature Analysis with BorutaShap

To quantify the relative importance of each feature for determining color class, we implemented BorutaShap, a wrapper for random forest (RF) ML algorithms, using Python.^59^ This package combines feature selection using the Boruta algorithm^59^ with Shapley additive explanations (SHAP).^60^ BorutaShap assigns each feature a maximum importance score compared to shadow attributes (MISA). Because BorutaShap is compatible with decision tree-based models, including RF, rather than SVM classifiers, we first verified that 1v1 RF classifiers perform well for Ag*_N_*-DNA color class discrimination. Figure S8 shows that 10-fold cross-validation scores for an ensemble of RF classifiers are comparable to the scores for the SVM-based model (Figure 3b). Out-of-bag errors for the RFs were found to be minimized using 100 decision trees in each RF, with default settings for all other parameters. To score features by importance for each 1v1 color class pair, regardless of class imbalance for that pair, we performed BorutaShap 10 times, with 10 distinct subsamples on each 1v1 classifier. The average MISA for each 1v1 classifier was computed, and any feature with a higher average MISA than the highest scoring shadow feature was selected as an important feature. An exception was made for any 1v1 pair containing NIR. With far fewer NIR sequences, subsampling to balance class size results in significant standard deviations of average MISA. Thus, for the NIR classifiers, features within one standard deviation of average MISA of the maximum shadow feature were selected as important. MISA scores are provided in Supporting Data 3.

The net importance score (*NIS*) is defined in Supporting Note 1. *NIS* is computed by either adding an importance score if the staple feature occurs more frequently in the specific color class than its 1v1 pair or subtracting the score if the motif occurs less frequently in the color class.

### Sequence Design

DNA template sequences for Green, Far Red, and NIR color classes were selected using the SVM ensemble architecture trained on the full data library (without reserving data for cross-validation) to screen all possible 4^10^ 10-base DNA sequences. We use all 144 staple features because SVMs regularized using the L1 norm naturally perform feature selection. For each 1v1 pair of color classes, the prediction probabilities of the 10 SVMs for that color class pair were averaged (capturing variation due to the distinct random training data subsamples). Then the minimum average prediction probability among the 1v1 classifiers for the target color class was assigned as a score for that sequence (i.e., to establish the Green score we compare average scores for Dark vs Green, Green vs Red, Green vs Far Red, and Green vs NIR). Sequences were ranked by score, and the top 124 sequences for each target color class were selected (this number enables the experiment to be carried out on one 384-well plate with 10 control DNA sequences for normalization to past training data).

### High-Throughput Synthesis and Characterization of Ag*_N_*-DNAs

Ag*_N_*-DNA synthesis was performed by robotic liquid handling on 384-well clear-bottom microplates. DNA was ordered with standard desalting in a 384-well plate from Integrated DNA Technologies, presuspended in DNase-free water at 40 μM. Ten wells contained a control oligomer known to produce bright Ag*_N_*-DNA products at 540 and 636 nm,^61^ which were used to normalize brightness to past experiments. DNA was mixed via pipetting with an aqueous solution of AgNO_3_ and NH_4_AcO (Sigma-Aldrich), pH 7, in the 384-well clear-bottom microplate. After 18 min, silver–DNA solutions were reduced by a freshly prepared solution of NaBH_4_ in H_2_O. Finally, the microplate was centrifuged at low speed for < 60 s to remove any small bubbles in microplate wells. Final stoichiometries were selected to match conditions used for training data collection (20 μM DNA, 100 μM AgNO_3_, and 50 μM NaBH_4_ for measurements in the visible spectrum^35^ and 20 μM DNA, 140 μM AgNO_3_, and 70 μM NaBH_4_ for NIR measurements,^24^ with 10 mM NH_4_OAc in both cases). The well plate was stored in the dark at 4 °C and measured 7 days after synthesis.

Fluorescence emission spectra from 400 to 850 nm were collected using a Tecan Spark instrument. A Tecan Infinite 200 Pro instrument equipped with a custom InGaAs femtowatt PIN photodetector (Newport) was used to measure fluorescence emission in the 675–1325 nm range, using 50 nm bandpass filters (Edmund Optics). Fluorescence measurements were corrected for detector spectral responsivity.^45^ On both plate readers, 260 nm light was used to universally excite all Ag*_N_*-DNAs, allowing rapid screening of all fluorescent products with a single excitation wavelength.^62^

### High-Throughput Spectral Analysis

To extract peak wavelength, λ_p_, and fluorescence brightness, in the 400–850 nm range, each fluorescence spectrum collected on the Tecan Spark instrument was fitted to a sum of one to three Gaussians as a function of energy. Fluorescence brightnesses of spectra were normalized using a control Ag*_N_*-DNA to enable direct comparison of brightness and λ_p_ among all samples (details in past works^35,39^ and the Supporting Information). Fluorescence measurements acquired on the custom NIR plate reader were characterized using a custom script to identify NIR peaks and calculate peak brightness and λ_p_, as described in Supporting Note 2 in the Supporting Information.

DNA sequence design is considered successful if the designed DNA strand produces a bright Ag*_N_*-DNA product of the correct color class. Because no direct comparison of fluorescence intensity among Green, Red, and Far Red brightness and NIR brightness was available for the training data library used here, and because our training data assigned DNA sequences to the NIR class if a NIR peak was reported by Swasey et al.,^24^ regardless of other detected peaks, we separately considered occurrences of NIR peaks to most fairly compare designed sequences to the training data set. Specifically, for Green, Red, and Far Red peaks, a sequence’s color class was assigned by the brightest fluorescent peak that was above the defined “brightness threshold” (details in the Supporting Information). Experimentally tested sequences that produced a bright NIR product were classified as NIR regardless of other bright color peaks present. If a sequence yielded both a NIR peak and additional bright Green, Red, and/or Far Red products, the sequence was classified as both NIR and as the brightest associated Green, Red, or Far Red fluorescent color. By this method, Green and Far Red sequence design was successful if the brightest product corresponded to the target color class, and NIR sequence design was successful if any bright NIR peak was measured (while not omitting information about peaks formed in other color classes). Full details are provided in Supporting Note 2 in the Supporting Information.

Fractional class composition of each color class for training data and designed sequences is given in Figure S13. Distributions of λ_p_ for DNA templates designed for Green, Far Red, and NIR color classes are given in Figure S14, and experimentally measured λ_p_ and fluorescence brightness are provided for all sequences in Supporting Data 2.

## Notes

Machine learning code and associated training data are available for download at https://github.com/copplab/Ag-DNA-design.

## Supporting Information Available

Experimental methods and data processing details, computational methods for *k*-means clustering, class definitions, one-hot encoding, and SVM parameters, results of *k*-means clustering; heat maps of average 10-fold cross-validation accuracies of ML classifier ensembles, average cross-validation accuracies for SVMs with truncated feature vectors, definition of net importance score and all values of these scores, additional figures of experimentally measured color distributions for designed sequences, and details to accompany supporting data tables (PDF)
Machine learning code and associated training data: Training_Dataset.xlxs (XLSX)
Results.xlxs (XLSX)
Boruta_scores.xlxs (XLSX)
