# Supplementary material for: Chemistry-Informed Machine Learning Enables Discovery of DNA-Stabilized Silver Nanoclusters with Near-Infrared Fluorescence
Source: ACS Nano. 2022 Sep 20;16(10):16322–31. doi: 10.1021/acsnano.2c05390 (PMC9620400; doi:10.1021/acsnano.2c05390)
Supplement: Supplementary file 1 — nn2c05390_si_001.pdf [file nn2c05390_si_001.pdf]

Supporting Information

Chemistry-informed machine learning enables discovery of DNA-stabilized silver nanoclusters with near-infrared fluorescence

Peter Mastracco,[a] Anna Gonzàlez-Rosell,[a] Joshua Evans,[b] Petko Bogdanov,[c] Stacy M. Copp, [a,d,e*]

[a] *Department of Materials Science and Engineering, University of California, Irvine, CA 92697, USA*

[b] *Chaffey College, Rancho Cucamonga, CA 91737, USA*

[c] *Department of Computer Science, University at Albany, SUNY, Albany, NY 12222, USA*

[d] *Department of Physics and Astronomy, University of California, Irvine, CA 92697, USA*

[e] *Department of Chemical and Biomolecular Engineering, University of California, Irvine, CA 92697, USA*

*Correspondence to stacy.copp@uci.edu

Table of Contents

1. Experimental Methods ____ 2
    1.1 High-throughput Ag_N-DNA synthesis ____ 2
    1.2 Experimental spectroscopy and data processing ____ 2
2. Computational Methods ____ 3
    2.1 k-means clustering **Figure S1** ____ 4
    2.2 Definitions of intensity thresholds, **Figure S2** ____ 4
    2.3 Generation of training data classes ____ 5
    2.4 Example of one-hot encoding, **Figure S3** ____ 6
    2.5 Machine learning parameters, **Figure S4** ____ 6
    2.6 Cross-validation heatmaps, **Figures S5-S8** ____ 7
    2.7 Feature analysis, **Figure S9** ____ 10
    2.8 **Supporting Note 1**: Net importance score for features ____ 12
    2.9 Net importance scores, **Figure S10, S11** ____ 13
3. Experimental Validation of ML Model ____ 16
    3.1 **Supporting Note 2:** NIR Ag_N-DNA identification ____ 16
    3.2 Results: **Figures S12, S13, S14** ____ 18
4. Data Library Details for Supporting Data 1-3 ____ 21
5. References ____ 22

1. Experimental Methods

1.1 High-throughput Ag_N-DNA synthesis is performed with a Tecan Freedom Evo 150 robotic liquid handler equipped with a 96 MultiChannel Arm in 384 well microplates. DNA is ordered with standard desalting from Integrated DNA Technologies, pre-suspended in DNase-free water at 40 µM in 384 well plates. 10 wells contain a control oligomer 5'-TTCCCACCCACCCCGGCCCGTT-3' that produces two bright Ag_N-DNA products at 540 nm and 636 nm.[1] The green product's highly reproducible fluorescence intensity is used to normalize fluorescence brightness values to the experiments from which training data was taken (described elsewhere[2]). Normalization accounts for differences in plate reader sensitivities across the decade of past experiments and is described in past works.[2–5]

To synthesize Ag_N-DNAs, DNA is mixed via pipetting with an aqueous solution of $AgNO_3$ and NH_4AcO (Sigma Aldrich), pH 7 in low volume 384 well clear bottom polystyrene microplates with a nonbinding surface (Corning #3540). After 18 minutes, solutions are reduced by a freshly prepared solution of $NaBH_4$ in ultrapure H_2O, followed by mixing via pipetting. Finally, microplates are centrifuged at low speed for < 60 seconds to remove any small bubbles in microplate wells; these infrequent bubbles may scatter light and are problematic for quantitative spectroscopy. Final stoichiometries match conditions used for training data collection: 20 µM DNA, 100 µM $AgNO_3$, and 50 µM $NaBH_4$ for measurements in the visible spectrum and 20 µM DNA, 140 µM $AgNO_3$, and 70 µM $NaBH_4$ for NIR measurements (10 mM NH4OAc in both cases). Well plates are stored in the dark at 4 °C and measured 7 days after synthesis. Full experimental details are provided in freely available supporting information of past publications.[2,6]

1.2 Experimental spectroscopy and data processing. Fluorescence emission spectra in the 400-850 nm range are collected using a Tecan Spark, in 2 nm steps with 20 µs integration time and excitation centered at 280 nm to universally excite all products.[7] For fluorescence emission spectra collected on the Tecan Spark, the peak fluorescence emission wavelength, λ_p, and fluorescence brightness of each Ag_N-DNA product detected are determined by spectral fitting (described in detail previously[2]). To summarize briefly, a peak-finding routine implemented with a custom script determines the number of peaks in the spectrum, up to a maximum of three peaks. Then, the spectrum is fitted to a sum of Gaussians of the form $f_i(x) = a_0 + A_i * \exp\left(-(E - E_i)^2/{w_i}^2\right)$ where E is photon energy and E_i is the energy at the center of the Gaussian peak. a_0 is an offset due to background signal ("dark" spectrum). Fitting is performed with

constraints $A_i > 0$ and peak width $w_i > 0.05$ (avoids fitting to noise). Peak energies are converted to peak wavelength: $\lambda_p = hc/E_i$, where h is Planck's constant and c is the speed of light in vacuum. Fits are excluded as non-physical if λ_p is outside of the instrument detection window, and for extremely broad peaks, $w_i > 0.5$ eV, which can correspond to multiple fluorescent products. Finally, fits are verified by eye and corrected or, in the rare case that no reasonable fit is possible, the sequence is excluded from analysis. Peaks are then annotated by their peak wavelength, λ_p, and peak brightness, defined as $A = A_i * w_i$ (proportional to peak area). We report peak brightnesses that are normalized to previous experiments using the control Ag_N-DNA described in Section 1.1

Fluorescence emission data in the NIR spectral region are collected using a customized well plate reader described previously, with the same excitation source as the Tecan Spark.[8] This NIR plate reader is equipped with a custom InGaAs detector, whose output is digitized by an analog to digital converter (ADC). Because the software controlling the well plate reader's motor and excitation source is separate from the acquisition software for the ADC, a mixture of PbS quantum dots that emit across the entire NIR wavelength range (Sigma Aldrich) is used as an indicator of the time of measurement of the first well (A1) in the file recorded by the ADC. The entire plate is measured for each bandpass filter (50 nm bandpass filters, spanning a spectral range from 675 – 1,325 nm in 50 nm increments). The raw data (voltage as a function of time) is processed for each of the 384 wells using a custom script in Igor Pro (Wavemetrics), including correction for detector spectral responsivity. Finally, we reconstruct the fluorescence intensity of each well in 50 nm steps corresponding to each bandpass filter.

2. Computational Methods

2.1 k-means clustering

K-means clustering was implemented using the sci-kit learn in python. All peak wavelengths in our dataset were clustered into 4 distinct bins. The optimal number of clusters was determined using the elbow method, which identifies the inflection point in the plot of inertia as a function of the number of clusters to be the appropriate number of clusters in a dataset; this inflection point corresponds to the point beyond which adding additional clusters results in "diminishing returns," or begins to contribute less to the performance of the k-means clustering model (Fig. S1b). Using the elbow test, the point of inflection on the graph corresponds to four color classes, which aligns with the previously expected number of classes (note that the Dark class is inherently absent from

this k-means clustering). The centroids for the Green, Red, Far Red, and NIR classes were placed at 547, 637, 687, and 797 nm, respectively, represented by the colored dots in Figure S1. The boundaries or midway points between these four centroids are therefore at 592, 662, and 750 nm (*e.g.* the boundary between the centroids at 637 nm and 687 nm is at (687-637)/2 = 662 nm). These boundaries align with the wavelength cutoffs for the previously identified color classes and provided a starting point for finding an optimal upper wavelength cutoff for the NIR class. The centroid for the NIR class is likely to be shifted towards the Far Red class due to the artificial peak that forms around 750 nm and is a result of combining together two different fluorescence detectors (Methods).

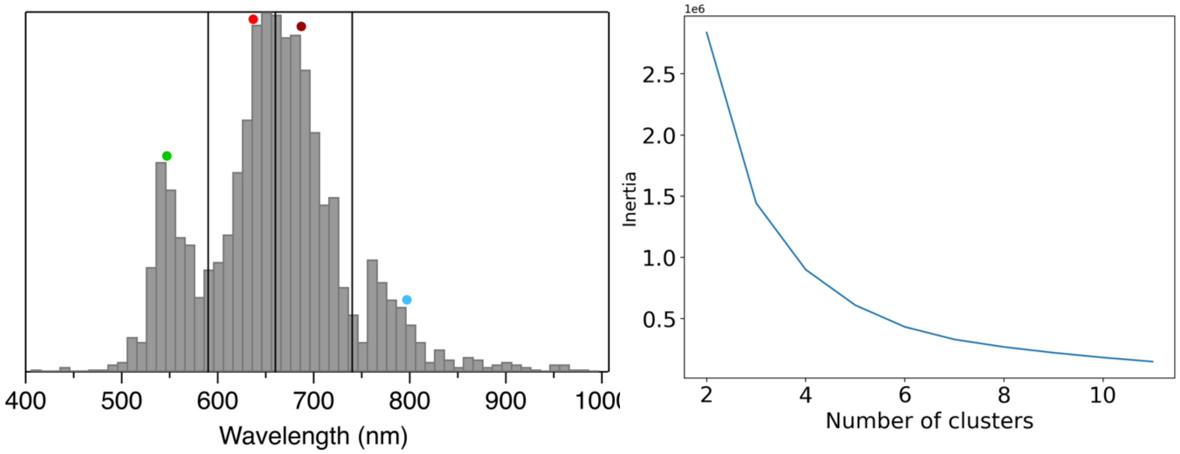

Figure S1. a) Distribution of peaks in the training data set, with the centroids of each class color class represented by colored dots and the cutoffs between each color class represented by vertical black lines (cutoffs defined to be the midway point between centroids). B) Elbow plot of inertia vs. number of clusters, indicating that the optimal number of clusters for the k-means clustering is four.

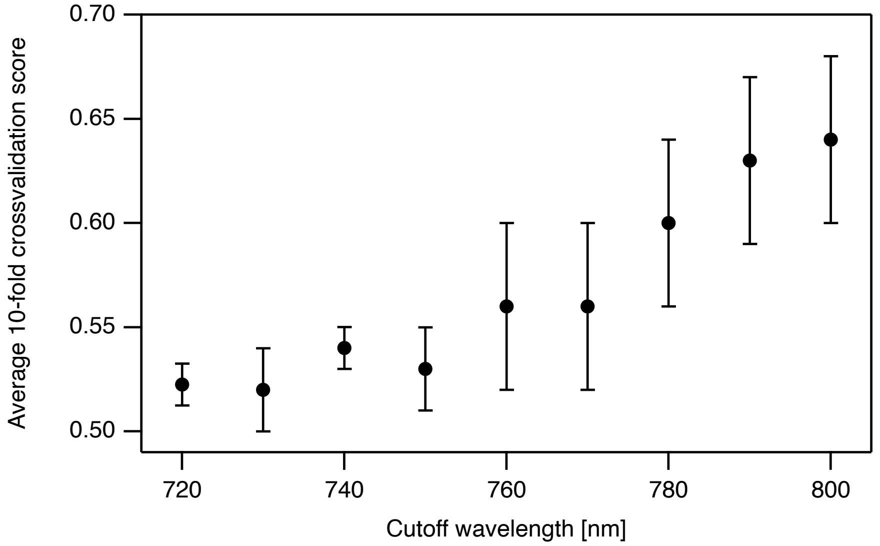

Figure S2. Average 10-fold cross-validation score of a set of 10 SVMs trained to discriminate Far Red vs. NIR, using sub-sampling to balance class sizes.

2.2 Definitions of intensity thresholds for Dark class and bright color classes

In our past work, sequences associated with the bottom 30% of integrated intensities were defined to be Dark, and sequences associated with the top 30% of integrated intensities were defined to be "bright."[4] The fluorescence intensity values corresponding to these initial definitions were preserved in later work to define the Dark, Green, Red, and Far Red classes.[2,5] To improve ML classification accuracies, here we refined the metrics used to define Dark (based on total integrated intensity) and bright (based on peak area $A = A_i * w_i$ of a fitted Gaussian, as described in 2.1) based on our past studies to better capture the sequence features that encode Ag_N-DNA color. To increase selectivity against Dark sequences by ML-enabled DNA ligand sequence design, we reduced the Dark integrated intensity threshold to 0.8 times the original definition, which increased average cross-validation scores for the majority of the 10 color class pairs. We also lowered the "brightness" threshold, *i.e.* the minimum *A* corresponding to a brightly fluorescent Ag_N-DNA product, as our past work found that many Green Ag_N-DNA products are "right on the cusp" of being classified as bright by the previous metrics. Lowering the brightness threshold to 0.8 times its prior value[2] increases the size of the Green class by 17%. Together, these changes also improve class balance by bolstering the size of the Green class and reducing the size of the Dark class.

2.3 Generation of training data classes

Definitions of color classes are described in detail in the main text. Here, we summarize how DNA sequences are sorted into these classes. All sequences and associated integrated intensities I_{int},

peak wavelengths λ_p, and peak areas A ("brightness" metrics) and NIR integrated intensities I_{nir} are provided in Supporting Data 1. These metrics have been described in detail in past studies.[2,4] Definitions are as follows:

- "Dark" defined as sequence whose $I_{int} < 0.8$
- "Green" as $\lambda_p < 580$ nm, A > 0.8, $I_{int} > 0.8$, and $I_{nir} <0.01$
- "Red" as 600 nm < λ_p < 660 nm, A > 0.8, $I_{int} > 0.8$, and $I_{nir} <0.01$
- "Far Red" as 660 nm < λ_p < 800 nm, A > 0.8, $I_{int} > 0.8$, and $I_{nir} <0.01$
- "NIR" as $\lambda_p > 800$ nm and $I_{nir} > 0.01$ or $I_{int} > 0.8$

Any sequences in the training data with normalized integrated intensity less than 0.8 and all peak areas less than 0.8 were excluded from the training data set. Furthermore, sequences associated with multiple bright fluorescence peaks in two color classes were excluded from training data. One exception to this rule is that any sequence with a brightly fluorescent NIR peak was placed into the NIR class regardless of any secondary peak, as there are very few identified sequences that produce NIR products, and it was necessary to include all NIR-forming DNA sequences to learn the NIR color-sequence correlations.

2.4 Example of one-hot encoding

T C C G G G T G G C

	1	2	3	4	5	6	7	8	9	10
A	0	0	0	0	0	0	0	0	0	0
C	0	1	1	0	0	0	0	0	0	1
G	0	0	0	1	1	1	0	1	1	0
T	1	0	0	0	0	0	1	0	0	0

Figure S3. Example of one "hot-encoded" positional features. The 4x10 matrix is converted into a 1x40 vector and provides a unique representation of each 10-base sequence.

2.5 Machine learning parameters. Support vector machines (SVMs) were implemented using scikit-learn, a machine learning package written in Python. An L1 regularization was used as the loss function, and the optimal parameterization value was found to be 0.1. The regularization parameter value (C) was selected by training the SVM with all 184 features and comparing the cross-validation accuracies as a function of C values ranging from 0.001 to 5 (Fig. S4). All other parameters for the SVM were set to default values of scikit-learn.

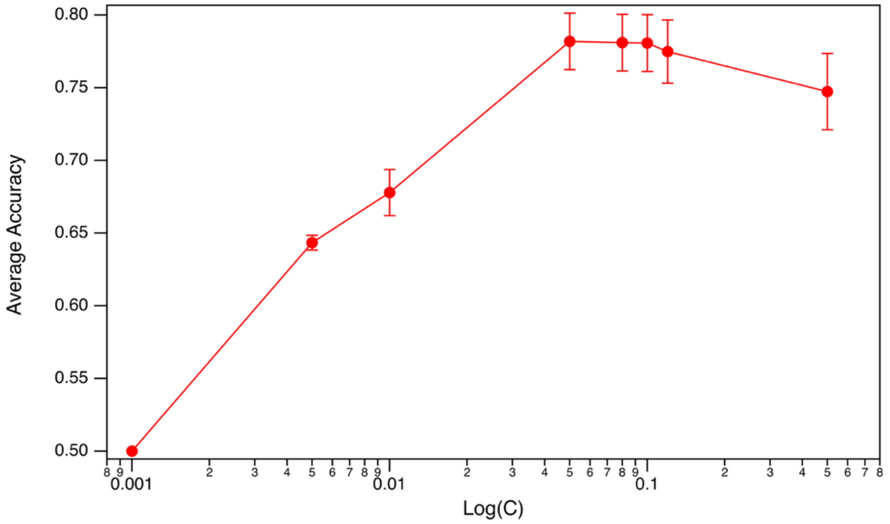

Figure S4. Average 10-fold cross-validation accuracy for all 1v1 SVMs in the ML model at different regularization parameters (C), *e.g.* the value for C = 0.1 is the average of all scores in Figure 3b.

2.6 Cross-validation heatmaps of 1v1 classifier ensembles. Random subsampling of the more abundant color class is used to balance class size when training each 1v1 classifier. Because random subsampling also alters the training data set, our model consists of a set of 10 1v1 classifiers per color class pair. To assess the accuracy of our model at mapping DNA sequence onto Ag_N-DNA color class, classifier performance was assessed by 10-fold cross-validation. This method splits training data into 10 folds, using 9 folds for training and one fold to assess classifier accuracy, and averages the accuracy from these 10 trained classifiers. For each 1v1 classifier, we performed 10-fold cross-validation process 100 times, to capture the variability in a large number of random data subsamples. The average and standard deviations of these cross-validation scores are reported in accuracy heatmaps (below).

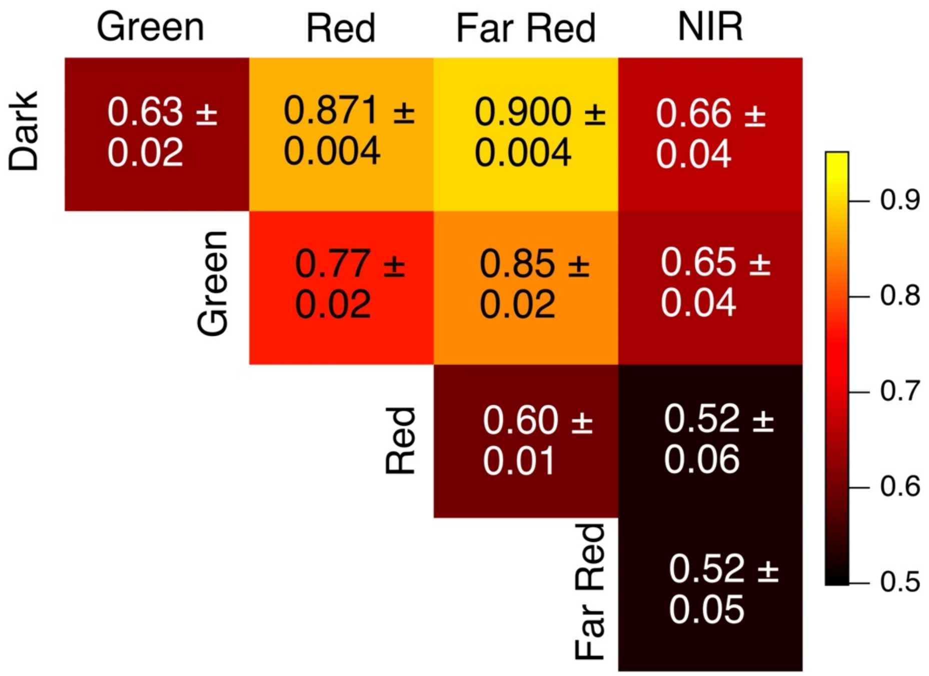

Figure S5. Accuracy heatmap for SVM-based model using only one-hot encoded positional features.

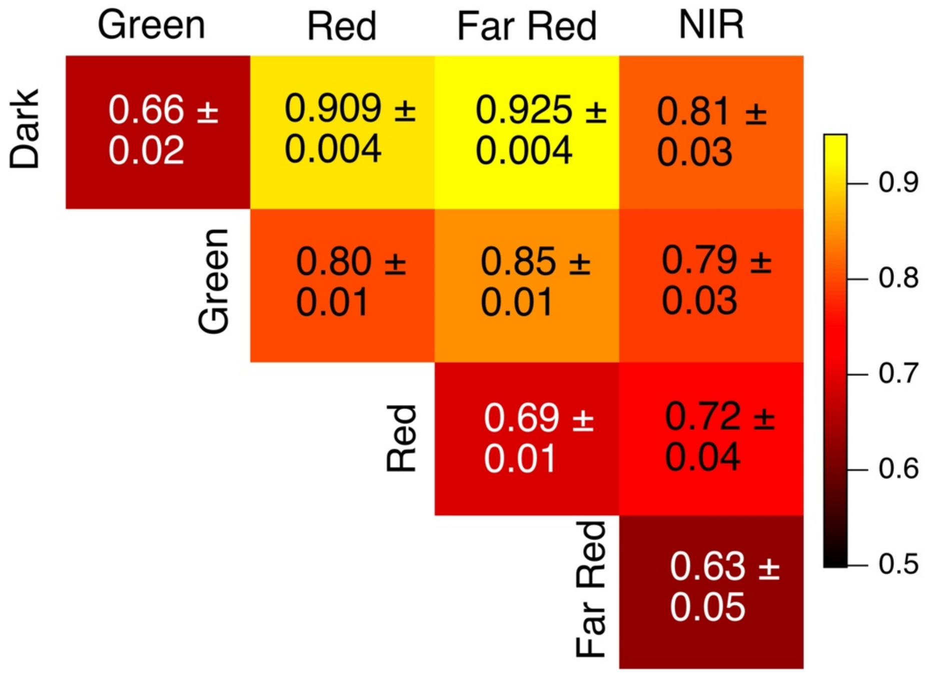

Figure S6. Accuracy heatmap for SVM-based model using only staple features. Note that Figure S6 is identical to Figure 3b and is provided here for easy comparison to Figures S5 and S7.

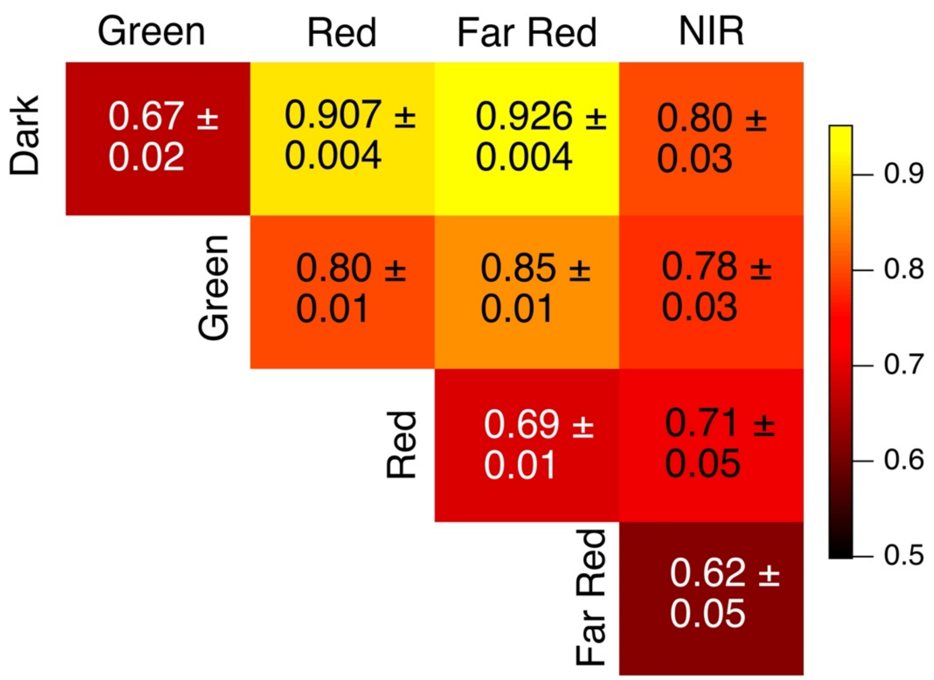

Figure S7. Accuracy heatmap for SVM-based model using both staple features and positional features.

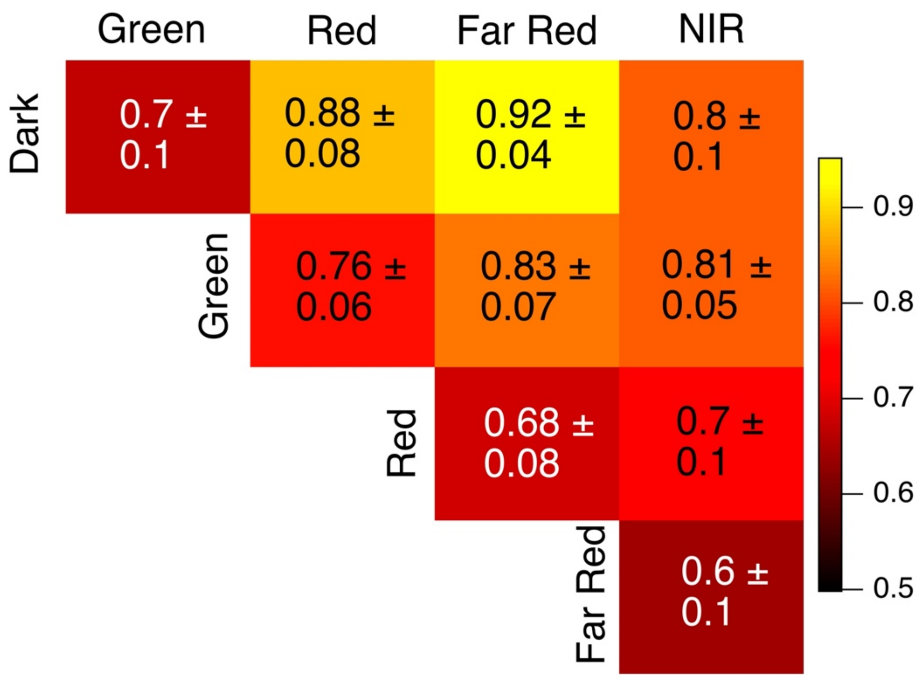

Figure S8. Accuracy heatmap for RF model using the top features selected for each classifier by BorutaShap.

2.7 Feature analysis

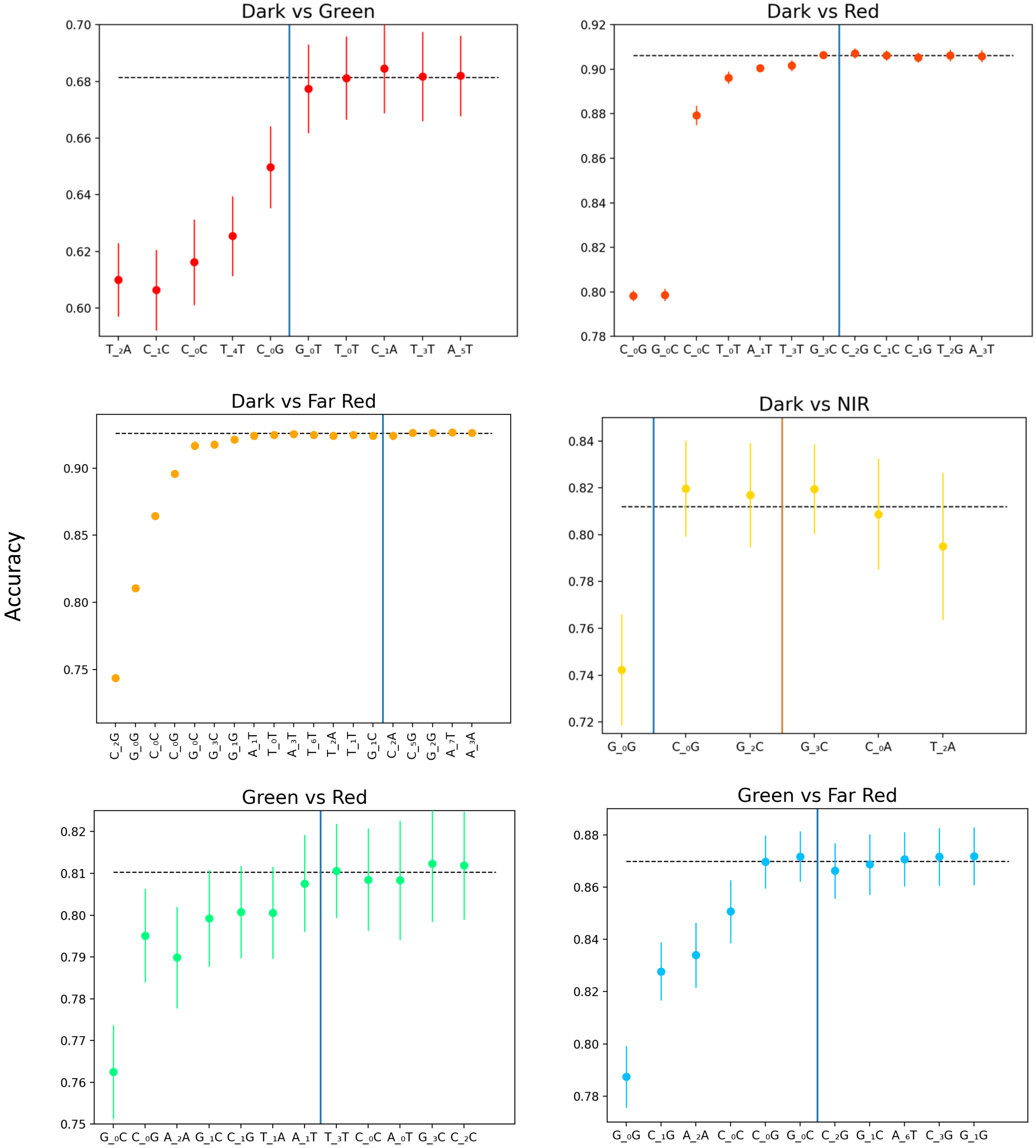

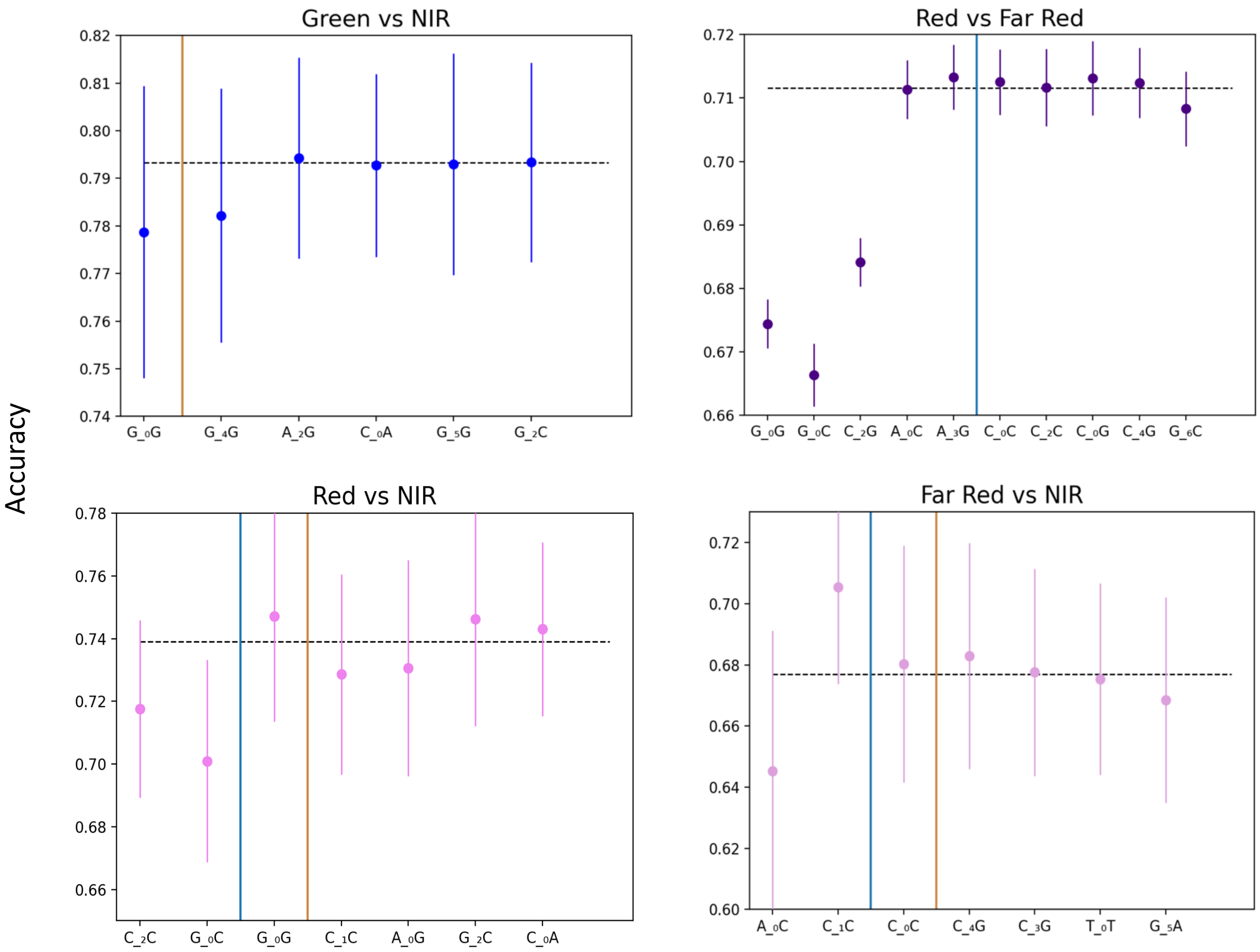

Figure S9. Average 10-fold cross-validation accuracies of each 1v1 SVM as a function of the number of staple features included in the feature vector. Features are ranked based on the average importance score assigned by BorutaShap; the horizontal axis lists features from left-to-right in order of this rank. Each cross-validation accuracy is calculated from a set of 10 SVMs trained on random subsamples of the training data and for feature vectors corresponding to all staple features listed to the left of the location on the horizontal axis. Features that scored greater than the maximum shadow feature (*i.e.* sufficiently greater than random) are to the left of the vertical blue lines. For color class pairs including the NIR class, features to the left of the brown line have average scores within a standard deviation of the maximum shadow feature.

2.8 Supporting Note 1: Definition of net importance score (*NIS*)

We implemented BorutaShap,[9] a wrapper for random forest (RF) ML algorithms, using Python.[10] BorutaShap assigns each feature a maximum importance score compared to shadow attributes (MISA). Shadow features are randomly generated and are therefore meaningless for classification. Thus, MISA provides a metric that can be used to compare the importance of real features to random noise. Because MISA are relevant to the comparison of two classes and not correlated to a single class, we defined a *Net Importance Score* to calculate the importance of a single motif for a single color class.

BorutaShap assigns an importance score to a specific staple feature *m* for each pair of color classes C_i and C_j, corresponding to a 1v1 classifier trained to discriminate C_i and C_j. Because there are five color classes (Dark, Green, Red, Far Red, and NIR), $i, j = 1, 2, 3, 4, 5$ and there exist ten BorutaShap-assigned importance scores I_{ij} ($j \neq i$ and $i, j = 1, 2, 3, 4, 5$) relevant to staple feature *m*. Consider the four BorutaShap-assigned importance scores for all color class pairs containing C_i. We define a quantity ε_{ij}, where $\varepsilon_{ij} = 1$ if the frequency of staple feature *m* is greater in C_i than in C_j and $\varepsilon_{ij} = -1$ if the frequency of staple feature *m* is smaller in C_i than C_j. Then, the net importance score for motif *m* in class C_i is defined by the following expression:

$$NIS_i = \sum_{j \neq i}^{5} I_{ij} \varepsilon_{ij} \qquad \text{(Supporting Equation 1)}$$

In this expression, the importance score I_{ij} is added to NIS_i if motif *m* occurs more frequently in sequences in C_i than in sequences C_j (accounting for class imbalance between C_i and C_j) because $\varepsilon_{ij} = 1$ supports that *m* is selective ***for*** C_i and against C_j. Vic versa, I_{ij} is subtracted from NIS_i if *m* occurs less frequently in sequences in C_i than in sequences C_j because $\varepsilon_{ij} = -1$ supports that *m* is selective ***against*** C_i and for C_j. For classifiers with no I_{ij} assigned for a staple feature *m*, the score of I_{ij} is assigned to a value of zero because it does not contribute to the overall score.

The *NIS* reported in Figure 4 and Figure S10 are calculated using I_{ij} that are averaged for ten applications of BorutaShap that are applied to each RF 1v1 classifier trained on ten different training data sets balanced by random subsampling of the more abundant class.

2.9 Net importance scores

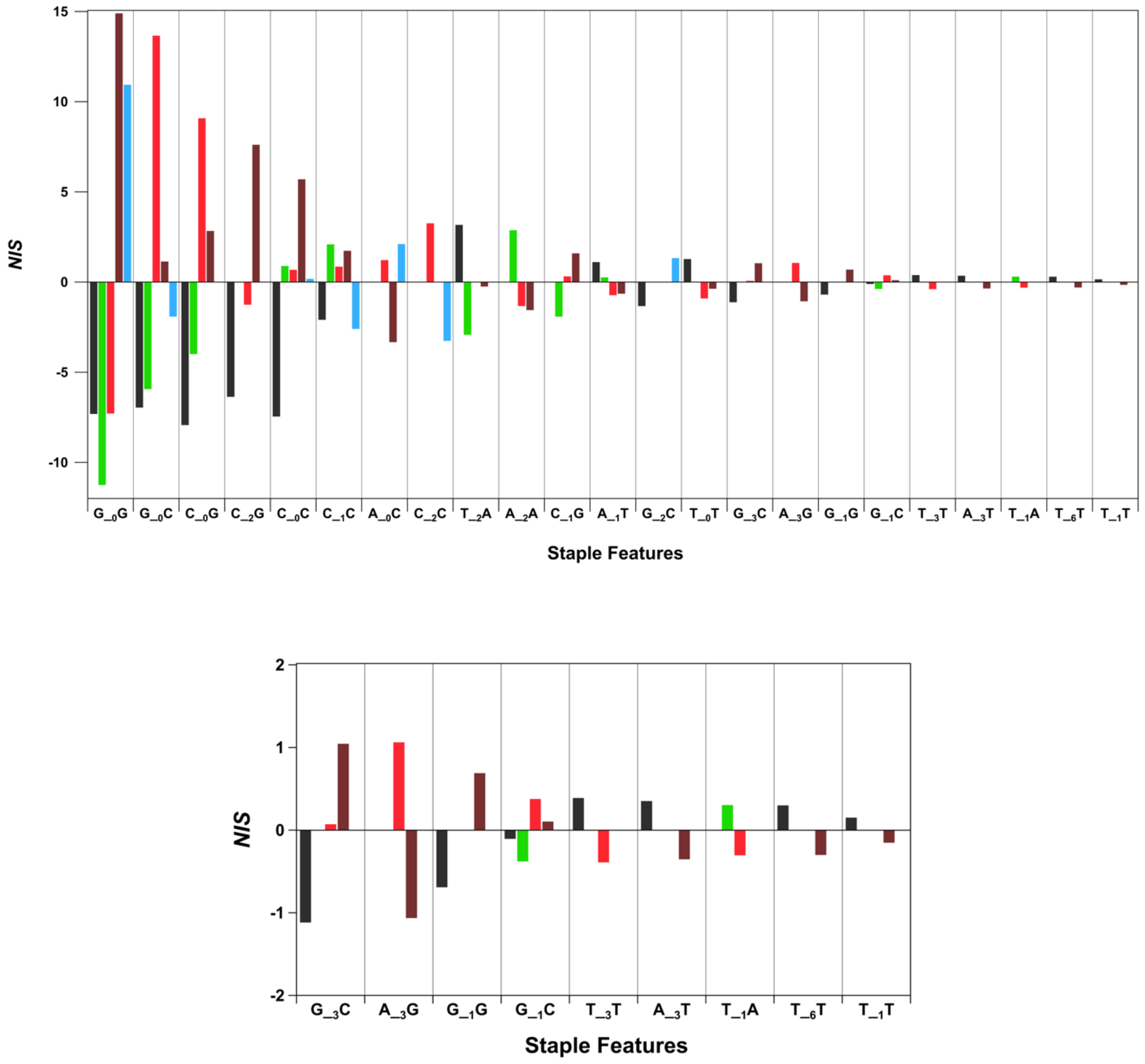

Figure S10. Net importance scores (*NIS*) for all 23 features whose average BorutaShap scores are greater than the maximum random shadow feature (for NIR-correlated features, features shown have average BorutaShap scores within one standard deviation of the maximum random shadow feature). *NIS* for Dark (black), Green (green), Red (red), Far Red (dark red), and NIR (blue). Bottom: zoom-in on *NIS* of low absolute magnitude from top graph.

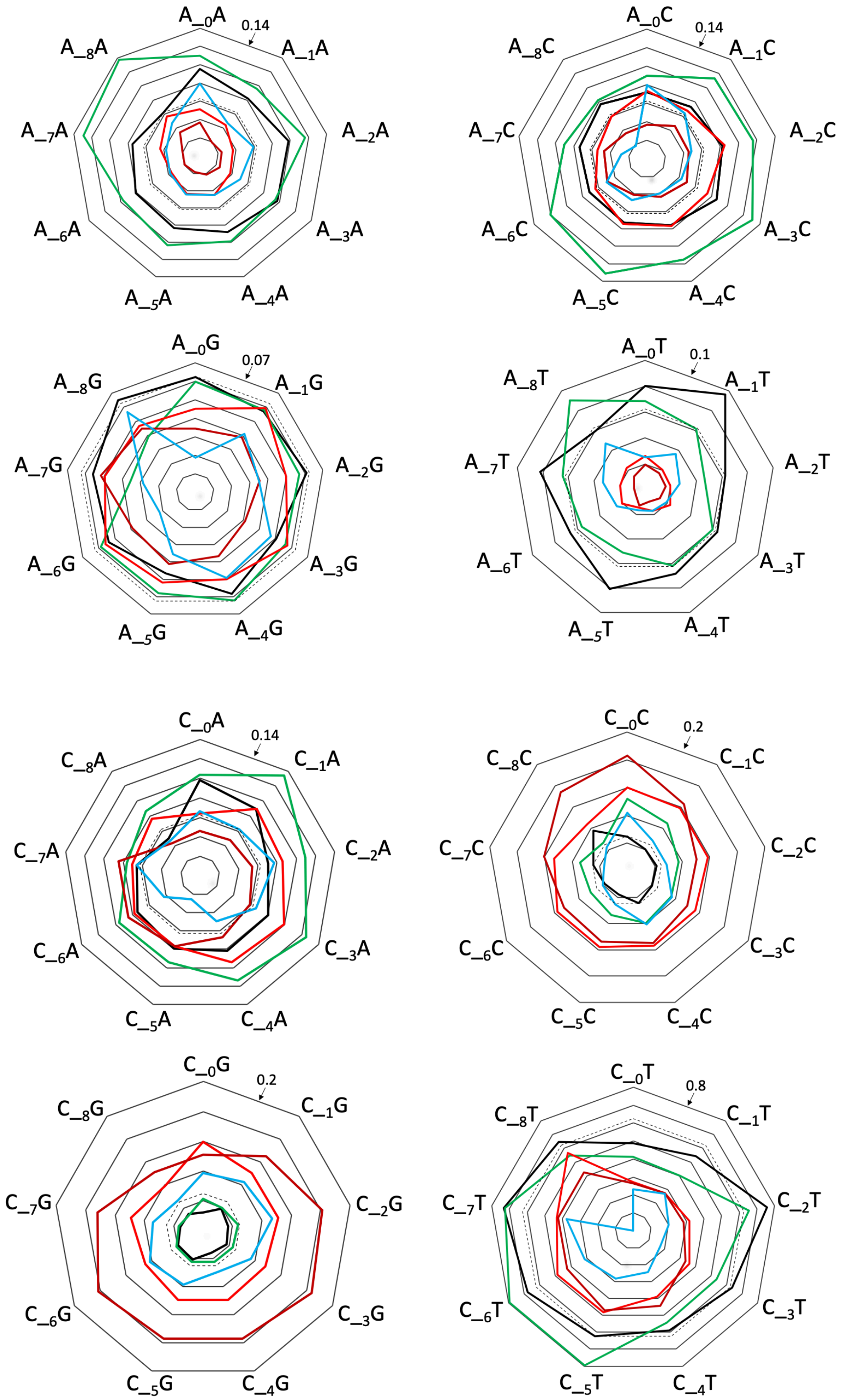

Figure S11. Radar graphs of normalized number of occurrences of staple features in Dark, Green, Red, Far Red, and NIR classes, as normalized by (total # of sequences)*(# of times the staple feature can occur in a 10-base sequence) Dark (black), Green (green), Red (red), Far Red (dark red), and NIR (blue). Dotted line represents frequency for all 10-base sequences. The center of the radar corresponds to zero, and the scale of each graph is indicated by the number pointing to the outermost line.

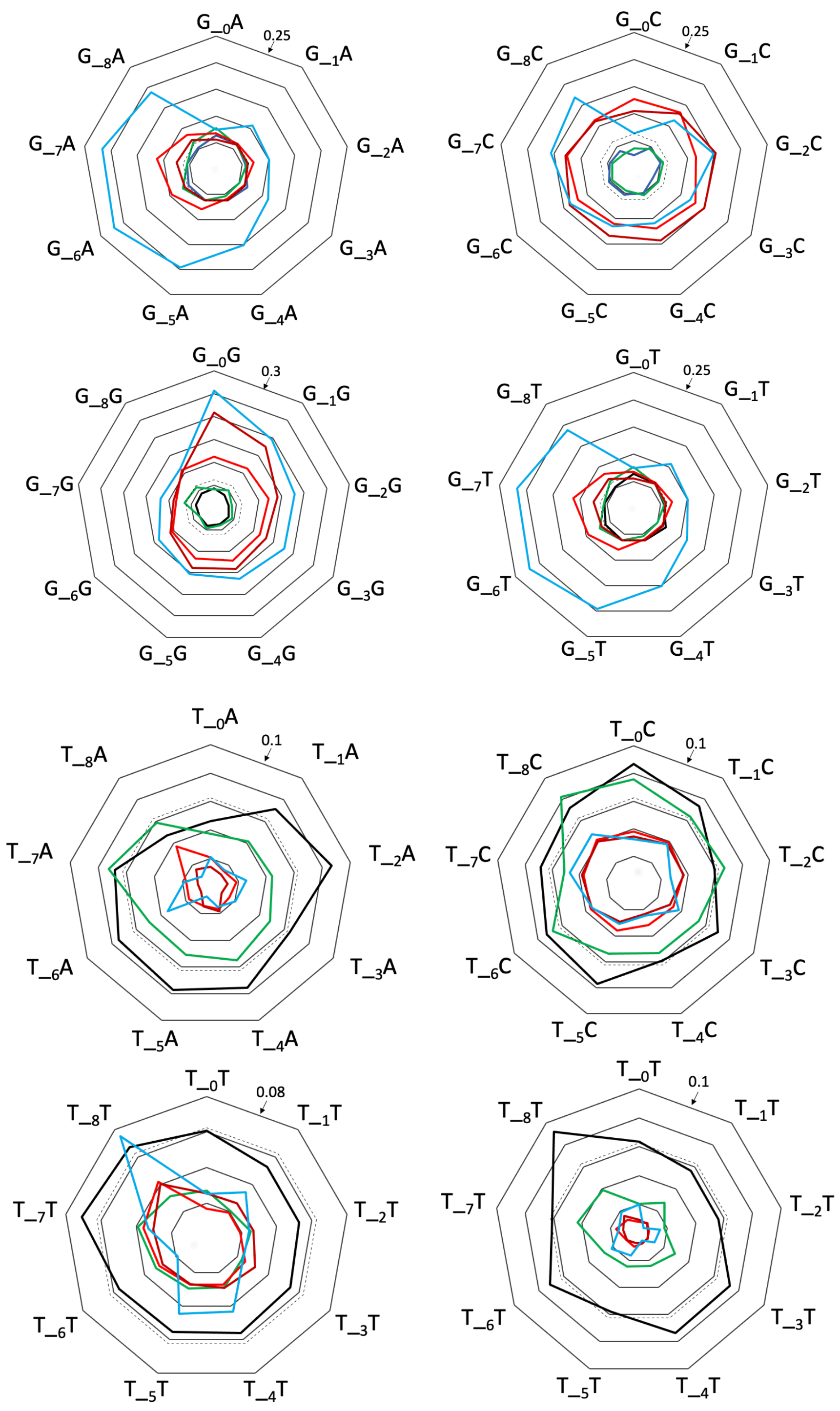

Figure S11. Continued, caption on previous page.

3. Experimental Validation of ML Model

3.1 Supplementary Note 2: NIR Ag_N-DNA identification

Our training data includes NIR Ag_N-DNAs reported by Swasey, *et al.,* which were identified by NIR integrated intensities $I_{nir} > 0.01$ in the 700 – 1,300 nm range,[6] as measured by voltage output of a InGaAs photoreceiver on a custom plate reader.[8] Here, we make two corrections to this method to ensure that brightly fluorescent NIR Ag_N-DNAs are properly identified and peak wavelength λ_p is accurately assigned. First, our plate reader has the same photoreceiver, but we cannot *a priori* assume the same spectral responsivity. To correct for potential variance in the responsivity of our well plate reader compared to the studies that produced our training data,[2,5,6] we normalize I_{nir} to past experiments by Swasey, *et al*. The ratio of total average integrated intensity of the control Ag_N-DNAs for experiments performed here to Swasey, *et al.,*[6] is 3.085. Therefore, we scale I_{nir} by this ratio, and the brightness threshold is increased to a new value of 3.085*0.01.

Our second correction is as follows. Swasey, *et al.*, assigned λ_p for Ag_N-DNAs measured on the NIR plate reader as the intensity-weighted wavelength average for all 50 nm bandpass filters from 700 – 1300 nm.[6] However, analysis of the spectra we collected on our NIR plate reader shows that many sequences have clearly defined NIR products with spectral "shoulders" from Far Red peaks detected for 50 nm bandpass filters at 700 nm and 750 nm that blueshift λ_p for some samples. In the visible spectral region, we account for sequences that produce multiple Ag_N-DNA products by multi-Gaussian spectral fitting, but this is infeasible with fluorescence intensities measured only every 50 nm. Therefore, we identify NIR peaks as follows. (1) Samples with increasing voltage signal for increasing bandpass filter wavelength above 800 nm are flagged. (2) Sequences whose integrated intensity in the range 850 nm to 1,300 nm is greater than I_{nir} = 3.085*0.01 are identified as "bright NIR." (Note that this metric of brightness is actually more stringent than used previously because we only consider intensities measured for 800 nm and above, excluding signals from 700 nm and 750 nm bandpass filters). This method identifies all but two of the NIR sequences that are identified by the simpler intensity-weighted wavelength average used by Swasey, *et al.*,[6] and finds an additional 15 sequences that are "multipeaked" with both Far Red and NIR peaks. (3) To more accurately capture peak wavelength in the NIR, we calculate λ_p as the local intensity weighted average of the bandpass filter with maximum signal (selected from filters 800 nm and above) and its two neighboring filters. Compared to the simpler

method used in the training data,[6] this method better approximates λ_p without blue-shifting due to a second peak in the Far Red region.

Experimentally tested sequences are assigned a color class as follows. Note that I_{int} is normalized to the control strand (Section 1.1). To be as clear as possible, we list I_{nir} as the normalized value (Section 3.1).

- "Dark" defined as sequence whose $I_{int} < 0.8$
- "Green" as $\lambda_p < 580$ nm, $A > 0.8$, $I_{int} > 0.8$, and $I_{nir} < 0.03085$
- "Red" as 600 nm $< \lambda_p <$ 660 nm, $A > 0.8$, $I_{int} > 0.8$, and $I_{nir} < 0.03085$
- "Far Red" as 660 nm $< \lambda_p <$ 800 nm, $A > 0.8$, $I_{int} > 0.8$, and $I_{nir} < 0.03085$
- "NIR" as $\lambda_p > 800$ nm and $I_{nir} > 0.03085$ (or $I_{int} > 0.8$ if a NIR peak is detected on the visible well plate reader)

For DNA sequences associated with more than one bright peak, color class is assigned as follows. If the sequence is associated with peaks in two or more of the Green, Red, and Far Red classes, the brightest peak in the Green to Far Red range is used to assign color class. This approach is rational because Green, Red, and Far Red peaks are detected using the same Tecan Spark plate reader. In contrast, NIR peaks are almost exclusively detected using the separate NIR plate reader, and no information was available in the training data to quantitatively compare brightnesses between the data we have reported[2,4,5] and NIR products reported by Swasey, *et al*, with this different well plate reader.[6] Therefore, we assign a sequence to two color classes if a NIR peak and a Green/Red/Far Red peak is detected. This approach is necessary to compare our designed sequences to those available in the training data.

Supporting Data 2 lists all designed sequences with measured normalized integrated intensities I_{int}, peak wavelengths λ_p, normalized peak areas A ("brightness" metrics), and normalized NIR integrated intensities I_{nir}. These values are used to generate Figures 5, S13, S14.

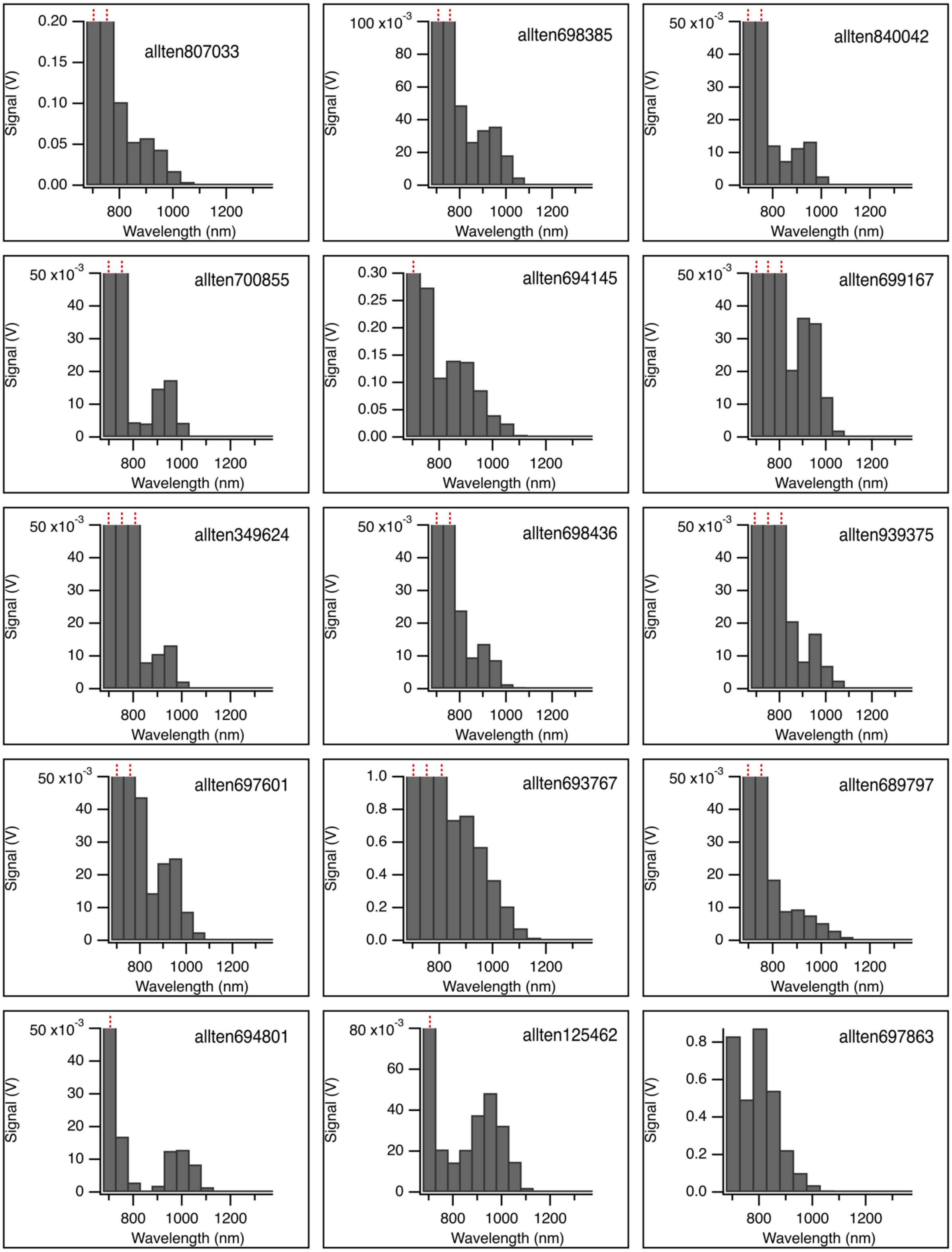

Figure S12. DNA strands that exhibit both Far Red and NIR and Far Red peaks. Red dots above bars for 700-800 nm indicate signals that extend above the y-axis range shown.

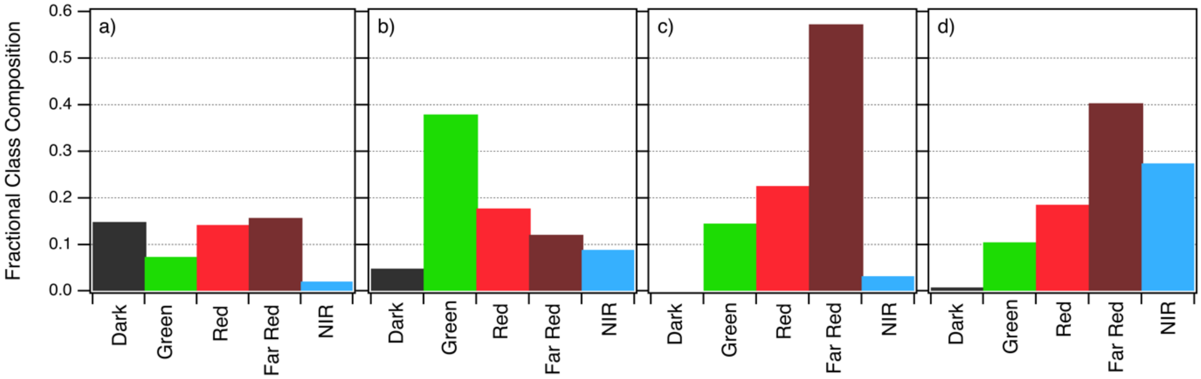

Figure S13. Fractional composition of each class for **a)** the “pristine training data” of 1,443 sequences, **b)** Green-designed sequences, **c)** Far Red-designed sequences, and **d)** NIR-designed sequences.

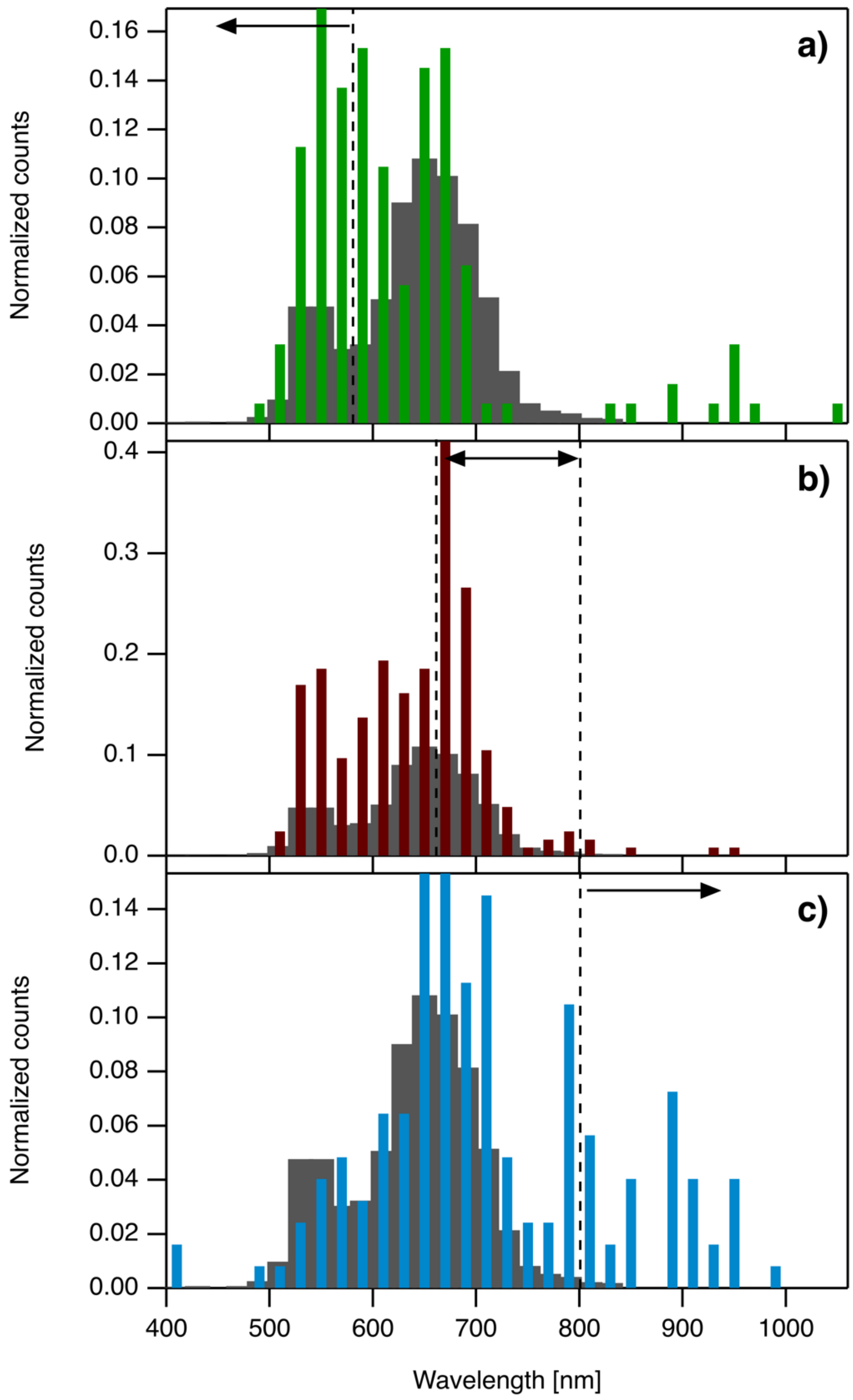

Figure S14. Probability density of bright peak wavelengths of the entire training dataset (black) as compared to the distribution of all experimentally measured bright peaks for predicted a) Green sequences b) Far Red sequences c) and NIR sequences. Note that if two or more bright peaks are detected for a single DNA sequence, all of these bright peaks are represented in this histogram.

4. Data Library Information

This section describes the associated Supporting Data Files for the training data used in this study and for the experimental tests of the developed ML model.

4.1 Supporting Data 1: Training Dataset

Training_Dataset.xlsx contains data for 2,661 sequences (Column A), aggregated from past studies,[2–6] with experimentally measured values for normalized total integrated intensity (Norm I) and the peak wavelength position(s) (peak 1, peak 2, peak 3) and associated normalized peak areas ("brightness") measured using a UV-Vis spectrometer. Additionally, sequences that were studied using high-throughput NIR spectroscopy[6] have information listed in the NIR bright peak and NIR normalized spectroscopy columns.

4.2 Supporting Data 2: Results.xlsx lists experimental results for designed DNA ligand sequences. The file contains sequences with their associated experimentally measured values for normalized total integrated intensity (Norm I) and the peak wavelength position(s) (peak 1, peak 2, peak 3) and associated normalized peak areas ("brightness") for each peak (Peak 1 Norm area, Peak 2 Norm area, Peak 3 Norm area), as measured using the Tecan Spark. NIR Norm Intensity is the normalized intensity identified using the NIR well plate fluorimeter, and NIR Peak lists all NIR peaks above 750 nm peaks with a weighted average above 750 nm (range chosen because the detection range for the fluorimeter is from 675-1325 nm and any sequence with a peak less than 750 nm is better described by the Tecan Spark).

4.3 Supporting Data 3: Boruta Results

The average MISA for staple features for each 1v1 RF classifier are listed in Boruta.xlsx. Staple features are listed in decreasing order by the average MISA for each feature using 10 different subsamples. The average MISA ("average"), standard deviation ("stdev"), and the number of times the feature was selected using different subsamples ("occurrences") are all listed.

5. References

(1) Cerretani, C.; Vosch, T. Switchable Dual-Emissive DNA-Stabilized Silver Nanoclusters. *ACS Omega* **2019**, *4* (4), 7895–7902. https://doi.org/10.1021/acsomega.9b00614.

(2) Copp, S. M.; Gorovits, A.; Swasey, S. M.; Gudibandi, S.; Bogdanov, P.; Gwinn, E. G. Fluorescence Color by Data-Driven Design of Genomic Silver Clusters. *ACS Nano* **2018**, *12* (8), 8240–8247. https://doi.org/10.1021/acsnano.8b03404.

(3) Copp, S. M.; Schultz, D.; Swasey, S.; Pavlovich, J.; Debord, M.; Chiu, A.; Olsson, K.; Gwinn, E. Magic Numbers in DNA-Stabilized Fluorescent Silver Clusters Lead to Magic Colors. *J. Phys. Chem. Lett.* **2014**, *5* (6), 959–963. https://doi.org/10.1021/jz500146q.

(4) Copp, S. M.; Bogdanov, P.; Debord, M.; Singh, A.; Gwinn, E. Base Motif Recognition and Design of DNA Templates for Fluorescent Silver Clusters by Machine Learning. *Adv. Mater.* **2014**, *26* (33), 5839–5845. https://doi.org/10.1002/adma.201401402.

(5) Copp, S. M.; Swasey, S. M.; Gorovits, A.; Bogdanov, P.; Gwinn, E. G. General Approach for Machine Learning-Aided Design of DNA-Stabilized Silver Clusters. *Chem. Mater.* **2020**, *32* (1), 430–437. https://doi.org/10.1021/acs.chemmater.9b04040.

(6) Swasey, S. M.; Copp, S. M.; Nicholson, H. C.; Gorovits, A.; Bogdanov, P.; Gwinn, E. G. High Throughput near Infrared Screening Discovers DNA-Templated Silver Clusters with Peak Fluorescence beyond 950 Nm. *Nanoscale* **2018**, *10*, 19701–19705. https://doi.org/http://dx.doi.org/10.1080/10428190802688509.

(7) O'Neill, P. R.; Gwinn, E. G.; Fygenson, D. K. UV Excitation of DNA Stabilized Ag Cluster Fluorescence via the DNA Bases. *J. Phys. Chem. C* **2011**, *115* (49), 24061–24066. https://doi.org/10.1021/jp206110r.

(8) Swasey, S. M.; Nicholson, H. C.; Copp, S. M.; Bogdanov, P.; Gorovits, A.; Gwinn, E. G. Adaptation of a Visible Wavelength Fluorescence Microplate Reader for Discovery of Near-Infrared Fluorescent Probes. *Rev. Sci. Instrum.* **2018**, *89* (9), 095111. https://doi.org/10.1063/1.5023258.

(9) Keany, E. BorutaShap : A wrapper feature selection method which combines the Boruta feature selection algorithm with Shapley values. https://doi.org/10.5281/zenodo.4247618.

(10) Kursa, M. B.; Rudnicki, W. R. Feature Selection with the Boruta Package. *JSS J. Stat. Softw.* **2010**, *36* (11), 1–13.
